# The Formin Diaphanous Regulates Myoblast Fusion through Actin Polymerization and Arp2/3 Regulation

**DOI:** 10.1371/journal.pgen.1005381

**Published:** 2015-08-21

**Authors:** Su Deng, Ingo Bothe, Mary K. Baylies

**Affiliations:** 1 Graduate Program in Physiology, Biophysics & Systems Biology, Weill Cornell Graduate School of Medical Sciences, Cornell University, New York, New York, United States of America; 2 Program in Developmental Biology, Sloan Kettering Institute, New York, New York, United States of America; New York University, UNITED STATES

## Abstract

The formation of multinucleated muscle cells through cell-cell fusion is a conserved process from fruit flies to humans. Numerous studies have shown the importance of Arp2/3, its regulators, and branched actin for the formation of an actin structure, the F-actin focus, at the fusion site. This F-actin focus forms the core of an invasive podosome-like structure that is required for myoblast fusion. In this study, we find that the formin Diaphanous (Dia), which nucleates and facilitates the elongation of actin filaments, is essential for *Drosophila* myoblast fusion. Following cell recognition and adhesion, Dia is enriched at the myoblast fusion site, concomitant with, and having the same dynamics as, the F-actin focus. Through analysis of Dia loss-of-function conditions using mutant alleles but particularly a dominant negative Dia transgene, we demonstrate that reduction in Dia activity in myoblasts leads to a fusion block. Significantly, no actin focus is detected, and neither branched actin regulators, SCAR or WASp, accumulate at the fusion site when Dia levels are reduced. Expression of constitutively active Dia also causes a fusion block that is associated with an increase in highly dynamic filopodia, altered actin turnover rates and F-actin distribution, and mislocalization of SCAR and WASp at the fusion site. Together our data indicate that Dia plays two roles during invasive podosome formation at the fusion site: it dictates the level of linear F-actin polymerization, and it is required for appropriate branched actin polymerization via localization of SCAR and WASp. These studies provide new insight to the mechanisms of cell-cell fusion, the relationship between different regulators of actin polymerization, and invasive podosome formation that occurs in normal development and in disease.

## Author Summary

Muscle formation and homeostasis critically depend on fusion between myoblasts to create and maintain multinucleated muscle fibers. Despite the importance of this process, the mechanisms regulating myoblast fusion are not fully understood. Previous studies have shown that actin polymerization factor Arp2/3 plays a critical role during myoblast fusion. However, whether other actin regulators also play a role during fusion, and how they coordinate with Arp2/3 in controlling actin dynamics remain unclear. Taking advantage of the model organism, *Drosophila melanogaster*, which shares the conserved muscle fiber with mammals, we identify the formin Diaphanous (Dia), which polymerizes linear actin filaments, as essential for myoblast fusion. We show that Dia is present at the fusion site, and with a new dominant negative Dia allele, we demonstrate that Dia functions after myoblast recognition and adhesion, but upstream of Arp2/3. Moreover, using *dia* loss and gain of function experiments, we show that Dia regulates myoblast fusion by regulating actin dynamics and by localizing the Arp2/3 regulators, SCAR and WASp, to the fusion site. Our study thus identifies new regulatory factors during muscle formation. It also suggests mechanisms by which Dia and Arp2/3 activities are coordinated to regulate actin dynamics *in vivo* during development and homeostasis.

## Introduction

Actin filaments are major components of a cell’s dynamic cytoskeleton. The remodeling of actin networks controls cell autonomous behaviors, such as cell shape changes and intracellular trafficking **[1]**. Highly regulated actin remodeling is also required in intercellular processes, such cell-cell adhesion and cell-cell fusion. Cell-cell fusion of myoblasts gives rise to the functional unit of muscle, the multinucleated myofiber (**[2]**, reviewed in **[3]**). A series of conserved steps, including cell-cell recognition, adhesion, membrane alignment, membrane pore formation and cytoplasmic mixing, have been identified during myogenic cell fusion across species. Given its powerful genetic approaches, its optical tractability, and its simplicity, the *Drosophila* embryonic body wall musculature is an ideal system to study the mechanisms underlying these steps in myoblast fusion *in vivo*. In *Drosophila*, a multinucleated muscle fiber arises through the fusion of two types of myoblasts: a single Founder Cell (FC), which determines muscle identity by expressing a unique combination of transcription factors (**[4,5]**, reviewed in **[6,7]**), and multiple Fusion Competent Myoblasts (FCMs) (reviewed in **[8,9,10]**). Upon fusion, the nucleus of the FCM adopts the identity and transcriptional profile of the FC/Myotube (reviewed in **[11,12]**). As in vertebrates, fusion in *Drosophila* is an iterative process and in the fly embryo, the different individual muscles result from as few as 2 events to as many as 24 events **[4]**.

Recognition and adhesion between the FCs/Myotubes and FCMs is mediated by four transmembrane molecules belonging to the immunoglobulin superfamily: the FC/Myotube-specific proteins, Dumbfounded (Duf; also known as Kirre) and Roughest, and their binding partners on the FCMs, Sticks and Stones (Sns) and Hibris **[13,14,15,16]**. After bidirectional signaling via these transmembrane receptors, a fusogenic synapse is established between the FC/Myotube and FCM, and accumulations of filamentous actin (F-actin) are observed on the opposing sides of the fusion site **[17,18,19,20]**. On the FC/Myotube side, a thin sheath of F-actin is present. On the FCM side, the F-actin focus, which makes up the podosome-like, invasive structure (PLS), forms **[21]**. These enrichments of F-actin are highly dynamic and resolve prior to cytoplasmic mixing between the two cells **[19]**. F-actin accumulation and resolution at the fusion site suggest a functional role for actin during fusion. Supporting this role, genetic screens have identified a number of fusion mutants that map to genes involved in Arp2/3-based actin remodeling **[8,9,10,22]**. Arp2/3 is regulated by two nucleation-promoting factors (NPFs), SCAR/WAVE (WASp family verprolin-homologous protein) and WASp (Wiskott-Aldrich syndrome protein) **[23,24]**. Both SCAR and WASp activate Arp2/3 through simultaneous binding of actin and Arp2/3 **[24,25]**. During myoblast fusion, the stability, localization, and activity of SCAR are regulated by the WAVE complex member, Kette (Nap1), and by the small GTPase Rac **[19,26,27]**, which is activated by the bipartite GEF, Myoblast city (Mbc; Dock180) and Elmo **[28,29]**. WASp is recruited to the fusion site via the WASp-interacting protein Solitary (Sltr) (also known as DWIP and Verprolin) and Blown fuse (Blow) **[30]**. The coordinated activities of SCAR and WASp lead to Arp2/3 activation and subsequently the formation of the F-actin focus, the invasive podosome, a fusion pore **[21]** and finally, cytoplasmic continuity **[17,31]**.

Arp2/3 has been shown to bind to an existing F-actin filament and nucleate a new branch. While Arp2/3 can nucleate F-actin filaments de novo, it does this slowly **[32]**. The presence of pre-existing filaments accelerates Arp2/3’s ability to form branched F-actin **[33]**. Formins, another group of actin regulators, complement the activity of Arp2/3 by generating linear actin filaments. Studies have revealed both collaborative and antagonistic relationships between members of the WAVE regulatory complex, Arp2/3, and formins. As examples, Abi, a member of the WAVE complex, has been shown to interact with the formin mDia1 to positively regulate cell-cell adhesion in tissue culture cells **[34]**. In contrast, mDia2, WAVE, and Arp2/3 have been shown to form a multimeric complex, which inhibits mDia2-dependent filopodium formation in cultured cells **[35]**. Arp2/3 and formins often act together in different in vivo contexts, including pseudocleavage furrow formation, cytokinesis, and filopodia formation in *Drosophila* primary neurons **[36,37]**. Particularly relevant for our studies in myoblast fusion are findings that, in cancer cells and macrophages, Arp2/3 and formins are required for the formation of podosomes, which resemble the invasive structure at the myoblast fusion site **[21,38,39]**. How Arp2/3 and formins interact to regulate actin dynamics in different *in vivo* contexts, particularly myoblast fusion, remains to be investigated.

The best characterized formin in *Drosophila* is Diaphanous (Dia), where it is critical for cellularization **[40,41]**, wound healing **[42,43]**, segmental groove formation **[44]**, dorsal closure **[45]**, and synapse growth **[46]**. Dia nucleates and elongates actin filaments through its FH1 and FH2 domains. The FH1 domain interacts with Profilin, which is an actin monomer-binding protein, to increase the local actin monomer concentration **[47,48]**. The FH2 domain binds to actin barbed ends, stabilizes newly formed actin dimers, and promotes the elongation of actin filaments **[36,37,49,50]**. The regulation of Dia activity involves autoinhibition and Rho GTPase-mediated activation. Dia autoinhibition relies on the interaction between its C-terminal DAD (Diaphanous Autoinhibitory Domain) region and the N-terminal DID (Diaphanous Inhibitory Domain) region **[51,52]**. The autoinhibited state of Dia is relieved when Rho-GTP binds to the N-terminal GBD (GTPase binding domain) region, thereby disrupting the DID-DAD interaction. Deletion of the Dia DAD domain inhibits the folding of Dia into the autoinhibitory conformation and results in constitutively active Dia **[45]**. Given the well-established role that Dia plays in actin regulation during development and the central position that actin plays in myoblast fusion, it is likely that Dia would play a role in myoblast fusion. However, no such role has been established.

Here we show that Dia-mediated F-actin polymerization is required for the formation of the podosome-like structure at the myoblast fusion site and is essential for the invasion of the FC/myotubes by the FCMs. We show that Dia is localized to the fusion site and there regulates F-actin polymerization: loss of Dia activity blocks fusion, and no actin focus forms at the fusion site. Failure in focus formation arises from a block in F-actin polymerization as well as an inability to accumulate the Arp2/3 NPFs, SCAR and WASp, at the fusion site. Gain of Dia activity also blocks fusion and significantly changes the organization of the F-actin focus through increased actin turnover, leading to an excess of non-invasive filopodia at the fusion site. We further demonstrate that Dia-mediated SCAR and WASp localization is disrupted at the fusion site under these conditions. Based on our findings, we propose that Dia is necessary for two activities at the fusion site: Dia initiates invasive podosome formation through formation of linear actin filaments. Dia activity is also required for the accumulation of the Arp2/3 NPFs, SCAR and WASp, whose activity subsequently leads to Arp2/3 activation at the fusion site. The concerted F-actin elongation and branching processes likely provide the structural integrity and the necessary force generation for the invasive podosome, which ultimately leads to cell-cell fusion.

## Results

### Diaphanous Is Localized to the Fusion Site during Myoblast Fusion

**Fig 1:**
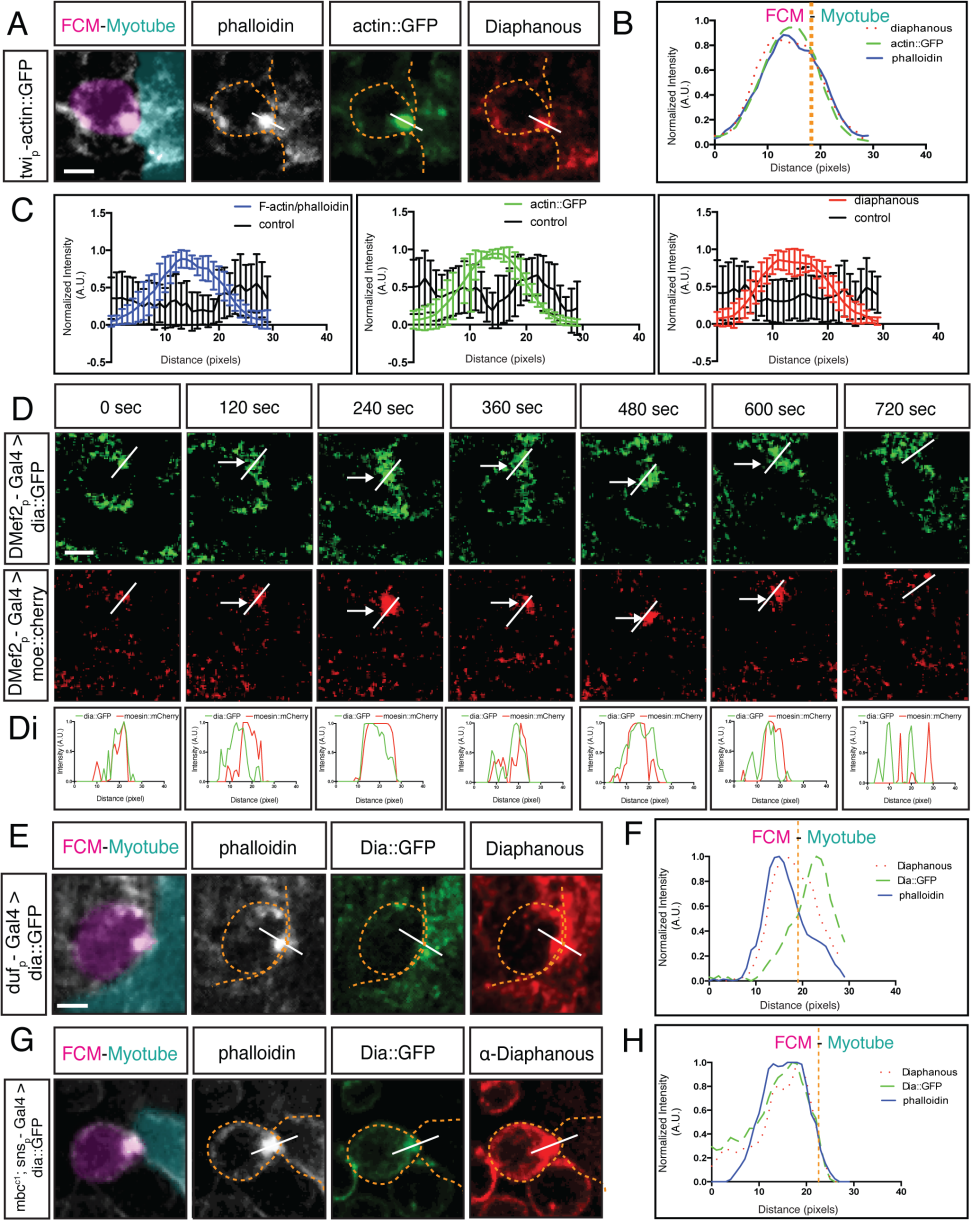
Diaphanous is localized to the fusion site. **A-D.** Dia colocalizes with the actin focus at the site of fusion. **A.** Fusing myoblast (FCM, false colored magenta in all Figures) and FC/Myotube (false colored turquoise in all Figures, see Methods and S1 Fig) in a stage 15 *twist-actin*::*GFP* embryo stained for F-actin (phalloidin, white) and antibodies against Dia (red) and GFP (green). Dia accumulates at the fusion site, colocalizing with the F-actin focus. **B.** Signal intensity plot confirms Dia enrichment with actin at the fusion site. Average fluorescence intensity measured across the F-actin foci as shown in A. A line of predetermined length was dropped across the fusion site; fluorescence intensity along the line was measured in different channels, normalized, and plotted (n = 10). See Methods and S2 Fig for details on intensity measurements and normalization. **C.** Fluorescent intensity curves with error bars for both control (n = 10) and proteins of interest (n = 10). **D.** Still images from a time-lapse series of a fusion event in a stage 14 embryo expressing Dia::GFP and Moesin::mCherry driven by *DMef2-Gal4* indicates that Dia::GFP has the same spatial and temporal pattern as actin at the fusion site. Moesin::mCherry (red) labels F-actin at the fusion site (arrows). **Di.** Signal intensity curve showing Dia::GFP (green) and Moesin::mCherry (red) colocalize during fusion at each time point. **E-H.** Dia is localized to the fusion site in both the FC/Myotube (turquoise) and FCM (magenta). Dia::GFP (green), phalloidin (white), endogenous Dia (red) **E.** *duf5*.*1-Gal4* driven Dia::GFP shows expression in myotubes/FCs in stage 14 embryos. Fusing FCM and myotube were captured before cytoplasmic mixing. Dia antibody staining (red) is present at the F-actin focus (phalloidin, white). FC driven Dia::GFP (green) expressed in FC/Myotubes partially overlaps with endogenous Dia at the fusion site. **F.** The signal intensity curve confirms that the peak of FC driven Dia::GFP partially overlaps with endogenous Dia. **G.** Stage 16 *sns-Gal4* driven Dia::GFP shows expression in *mbc* ^*C1*^ mutant FCMs, where fusion is blocked. Staining as in F. Dia enrichment is seen on FCM side with both Dia (red) and FCM driven Dia::GFP (green). **H.** Signal intensity curve confirms Dia::GFP and Dia overlap and are within the F-actin peak. Scale bar: 2.5μM

To investigate the role of Dia during myoblast fusion, we first examined its subcellular localization in fusing myoblasts (S1 Fig). The fusion site is identified by the presence of the F-actin focus **[19,21]**. Immunostaining revealed that Dia is present in the cytoplasm and cell cortex of myoblasts (S1 Fig) and accumulates at the fusion site between adhered myoblasts (Fig 1A and 1B). The specific accumulation of Dia at the fusion site was verified by quantification of Dia fluorescence intensity and comparison to phalloidin and Actin::GFP intensities (Fig 1B and 1C, n = 10; S2 Fig). Together, our analysis of fixed embryos indicates that Dia is enriched at the fusion site.

The F-actin focus is a dynamic structure that forms and subsequently resolves upon myoblast fusion. To determine whether Dia displays a similar profile, we used time-lapse analysis to compare the spatial and temporal dynamics of Dia and F-actin during fusion. Moesin::mCherry **[53]** and Dia::GFP **[45]** were expressed in myoblasts to label F-actin and Dia, respectively. Dia::GFP is reported to retain all Dia activity **[45]**. In myoblasts co-expressing both constructs, the lifetime of the F-actin focus was, on average, 16.8±6.9 minutes (n = 5), with a range from 9 to 25 min. This is comparable to expression of F-actin reporters alone **[19,54]**. We also confirmed that expression of these constructs under these conditions had no observable effects on muscle differentiation. Subsequent analysis of Dia::GFP and Moesin::mCherry revealed that Dia is present at the fusion site during F-actin focus formation and resolution (Fig 1D and 1Di). While Dia localizes to the cell cortex before and after a fusion event, it clearly accumulates at the fusion site coincident with the F-actin focus. Together, these data indicate that Dia becomes enriched at the fusion site with the same spatial and temporal dynamics as the F-actin focus.

Myoblast fusion is an asymmetric process in which the FCM produces a podosome-like structure that invades and promotes fusion with the FC/myotube. On the subcellular level, this asymmetry manifests in an uneven distribution of F-actin **[21]**. We therefore examined if Dia was also asymmetrically localized. To do this, we expressed Dia::GFP specifically in either FC/Myotubes or in FCMs and co-stained with the Dia antibody. By examining the overlap of exogenous and endogenous Dia signal, we can determine whether Dia is localized at the fusion site in one or both cell types.

We first expressed Dia::GFP specifically in FCs/Myotubes and examined Dia::GFP and Dia distribution. Before fusion pore formation and cytoplasm mixing, Dia::GFP could readily be detected only in the FC/Myotube. Dia antibody, in contrast, detected endogenous Dia in both the FC/Myotubes and FCMs. With this labeling approach, the Dia and Dia::GFP signals partially overlapped (Fig 1E). The partial colocalization between the FC/Myotube derived Dia::GFP and Dia antibody staining was confirmed by the separated peaks of the fluorescence intensity curves (Fig 1F). Thus, Dia is present in the FC/Myotube side during fusion, but this expression only constitutes a small portion of the total Dia enrichment at the fusion site.

Next, we examined the Dia accumulation on the FCM side. We expressed Dia::GFP specifically in FCMs, and assessed the localization of Dia::GFP and Dia. To prevent cytoplasmic mixing and the introduction of Dia::GFP into the FC/Myotube after fusion, Dia::GFP was expressed in FCMs of *mbc* mutant embryos, in which myoblast fusion is blocked prior to fusion pore formation **[55,56]**. Hence, no cytoplasmic exchange occurs between FCM and FC in *mbc* mutants. In FCMs, Dia::GFP accumulated at the fusion site (Fig 1G) and colocalized with endogenous Dia and the F-actin focus. The colocalization of Dia::GFP, Dia, and F-actin was confirmed by the overlapping fluorescence intensity curves (Fig 1H). These findings demonstrate that Dia enrichment at the fusion site, like F-actin, occurs primarily in the FCM, whereas in FC, only a thin layer of Dia is detected along the fusion interface.

### Dia Localization Is Dependent on FC/FCM Recognition and Adhesion, but Is Independent of Arp2/3 Dependent Actin Regulation

**Fig 2:**
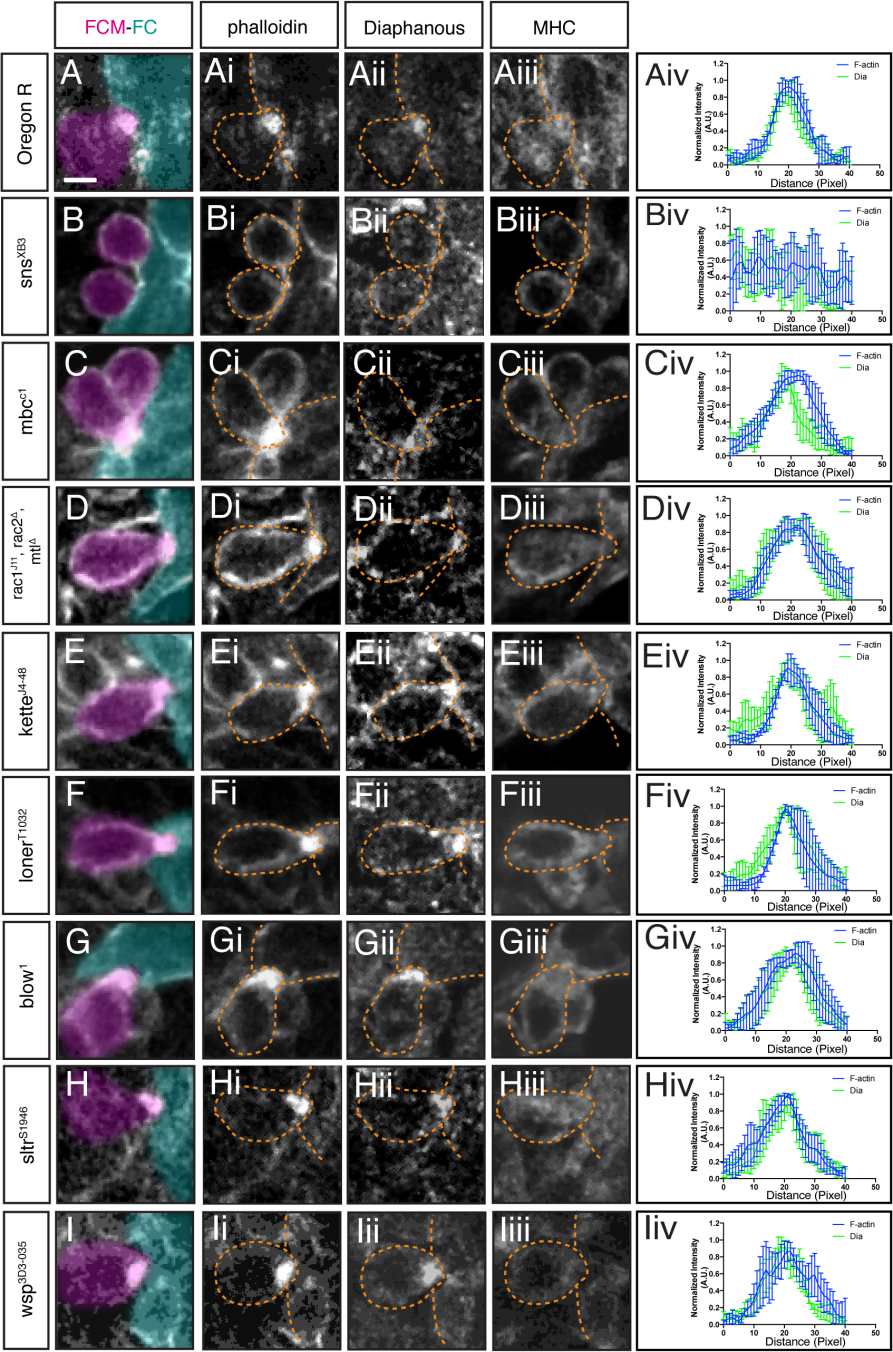
Dia localization at the fusion site is dependent on FC/FCM recognition and adhesion, but independent of regulators of Arp2/3. Stage 15 embryos stained with phalloidin (**i.**), antibodies against Dia (**ii.**), and Myosin Heavy Chain (**iii.**, MHC). Phalloidin labels F-actin (focus and sheath) at the fusion site; MHC identifies myoblasts. FCM (magenta) and FC/Myotube (turquoise). **A-iv.** Dia localization in FCM and FC/myotube in a wild-type embryo during myoblast fusion. Dia accumulates at the fusion site. The averaged fluorescence intensity curve (Aiv, n = 5) in wild-type embryos confirms Dia colocalization with actin. **B-iv.** In *sns* mutants, no F-actin focus is formed and no specific accumulation of actin or Dia are observed. Average fluorescence intensity curve of *sns* mutant embryos (Biv, n = 5) supports that Dia does not accumulate at the fusion site and is cytoplasmic. **C-E-iv.** In *rac*, *mbc*, and *kette* mutants, SCAR activity is lost, an enlarged focus is observed at the fusion site, and Dia is enriched at the fusion site. Fluorescence intensity curves confirm Dia and actin colocalization in *rac* (Civ), *mbc* (Div), and *kette* (Eiv) mutants (n = 5/genotype). **F-iv.** In *loner* mutant embryos, Dia accumulates at the F-actin focus, as confirmed by the fluorescence intensity curves (n = 5). **G-I-iv.** In *blow*, *sltr(Dwip)* and *wsp* mutants, where WASp-mediated actin remodeling is lost, Dia accumulation at the fusion site is unaffected. Fluorescence intensity curves confirm the colocalization of Dia and F-actin in *blow* (Giv), *sltr(Dwip)* (Hiv), *and wsp* (Iiv) mutants (n = 5/genotype). Scale bar: 2.5μM.

Dia enrichment at the fusion site suggested a role for Dia in myoblast fusion. To determine where in the fusion pathway Dia could function, we examined Dia localization in mutants in which myoblast fusion is blocked (Fig 2; S1 Table; S2 Fig). In *sns* mutants, where FCM-FC recognition is disrupted and no F-actin focus forms, Dia did not accumulate at the fusion site, but showed diffuse localization in the cytoplasm (Fig 2B, S1 Table), suggesting that Dia functions downstream of cell recognition and adhesion during fusion. Embryos carrying mutations in genes that regulate SCAR activity—*rac*, *mbc* and *kette*—display an enlarged actin focus that does not resolve. In these mutants, Dia accumulation is largely unaffected in the examined actin foci (Fig 2C–2E; S1 Table). We next examined embryos mutant for genes that regulate WASp activity: mutants in *blow* and *wsp* show enlarged F-actin foci, whereas mutants in *sltr/Dwip/vrp*, show normal sized foci. Dia localization appeared unchanged in all these mutants (Fig 2G–2I; S1 Table). Mutants in *loner*, which encodes an ARF-GEF family member **[57]** display normal-sized actin foci. In *loner* mutants, Dia accumulated at the F-actin focus at the stalled fusion site (Fig 2F; S1 Table). Dia’s enrichment at the fusion site in all these mutant conditions was quantified by fluorescence intensity curves (Fig 2Aiv–2Iiv, n = 5/genotype; S2 Fig). Together these data indicate that Dia localization at the fusion site is dependent on FC/FCM recognition and adhesion, but appears to be independent of Arp2/3 actin regulation.

### Dia Loss of Function Leads to a Myoblast Fusion Block

The localization of Dia in fusion mutant embryos suggested a role for Dia downstream of FC/FCM recognition and adhesion. Hence, we examined muscle formation, and myoblast fusion in particular, in *dia* mutant embryos, using well-established *dia* alleles **[40,58]**. During *Drosophila* embryogenesis, Dia is required in numerous processes, including metaphase furrow organization during division, cellularization, pole cell formation **[40]**, segmental groove formation **[44]** and dorsal closure **[59]**. In zygotic *dia* ^*5*^ */dia* ^*5*^ and *dia* ^*2*^ */dia* ^*2*^ mutants, abnormalities in the muscle pattern were found, including insufficient fusion (detected by free myoblasts), missing muscles, muscle morphology changes, and muscle detachment from its tendon cell (S2 Table; S3A Fig). We quantified the level of myoblast fusion by counting the total number of nuclei in the four Lateral Transverse (LT) muscles/hemisegments in both *dia* ^*2*^ and *dia* ^*5*^ homozygous mutants. Using this approach, we found a reduction in fusion (fusion index: *dia* ^*2*^: 14.1±1.3, n = 21; *dia* ^*5*^: 21.5±1.3 n = 20; control: 27.8±0.3 n = 12; p<0.001; S3B Fig). These defects in the musculature could contribute to reduced viability of the *dia* ^*2*^ and *dia* ^*5*^ homozygous mutants, as less than 10% of *dia* ^*2*^ homozygous mutants and only 20% of *dia* ^*5*^ homozygous mutants hatched into larvae. While these data suggested a role for *dia* in myoblast fusion, we sought out genetic conditions that would enable us to study myoblast fusion in more detail. In particular, we wanted to: 1- eliminate the effects of loss of Dia’s function in the ectoderm. The ectoderm is known to impact muscle development **[6,60]**; 2- increase the number of embryos which have Dia’s function abrogated; and 3- increase, if possible, the level of fusion block when Dia’s function is reduced. The Gal4/UAS system **[61]** allows generation of embryos in which 100% of the embryos express the transgene and can have a phenotype rather than 25% that results from traditional genetic alleles. Pairing the mesoderm/muscle specific *Dmef2-GAL4* with an appropriate UAS-line would allow manipulation of Dia in the cell type and during the time period in which fusion occurs. Available UAS-DiaRNAi lines, however, did not prove effective in knocking down Dia function during embryonic muscle development (S2 Table). We thus generated dominant negative Dia (DiaDN::GFP) transgenic flies to reduce Dia activity specifically in the developing mesoderm/muscle during myoblast fusion.

**Fig 3:**
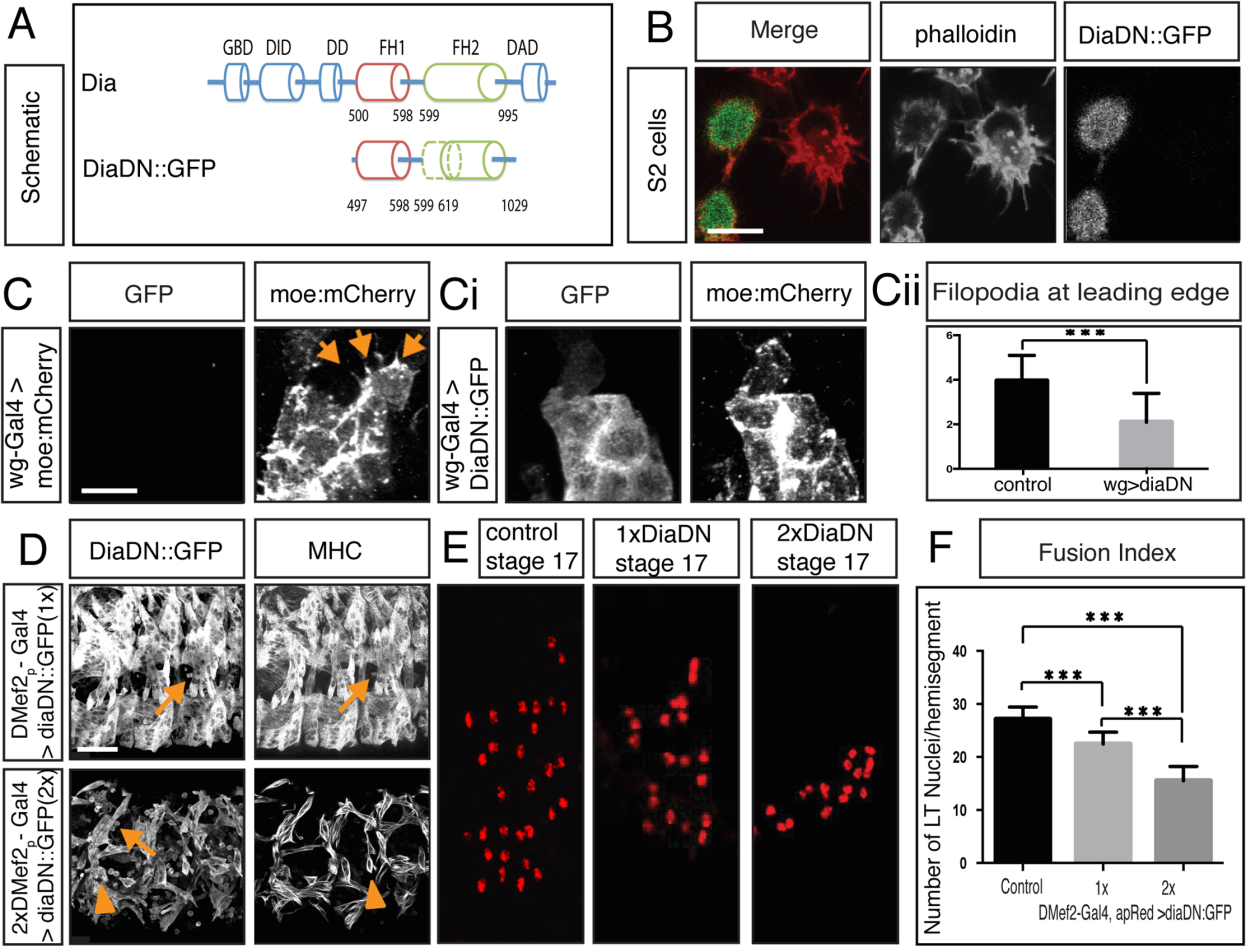
Diaphanous is required for myoblast fusion. **A.** Schematic diagram of Dia domain structure and a deletion construct that renders Dia dominant negative (DiaDN). DiaDN consists of the FH1 domain and a partially deleted FH2 domain; the deleted aa 750–770 in the FH2 domain is indicated by the dashed area. **B**. Expression of DiaDN reduces filopodia number in S2R+ cells. S2R+ cells that were transfected with DiaDN::GFP (green in merge, grey in single channel) have less filopodia-like, protrusive structures (phalloidin, red in merge, grey in single channel) relative to untransfected cells (n = 20, p<0.001). Scale bar: 10μM. This reduction was rescued by expression of Dia::GFP (S3C Fig). **C-Ci.** *UAS-diaDN*::*GFP* was expressed in leading edge cells using *wg-Gal4*. Moesin::mCherry was also expressed in leading edge cells to visualize actin. In stage 15, GFP-negative control cells, filopodia structures are seen (C, arrow). DiaDN::GFP significantly reduced filopodia formation (Ci). Scale bar: 2.5μM. **Cii.** Filopodium number was quantified in each wg-Gal4 expressing stripe. DiaDN significantly reduced filopodia formation in leading edge cells relative to control (2.1±1.29μM vs 3.95±1.15μM, p<0.001). **D.** Increasing DiaDN concentration in myoblasts through higher temperature and genetic copy number leads to an increased fusion block. Three hemisegments of a lateral view of a stage 16 embryos stained with GFP and MHC antibody are shown. Myoblast fusion is relatively normal in 1x*DMef2-Gal4>*1x*diaDN*::*GFP* embryos at 29°C (upper panel), with few free myoblasts (arrow). In 2x*DMef2-Gal4>*2x*diaDN*::*GFP* embryos (lower panel), a higher degree of fusion block (arrows) and muscle detachment (arrowheads) are observed. **E.** One hemisegment of stage 17 embryo showing apME-NLS::dsRed labeled nuclei in the four lateral transverse (LT) muscles: From left to right: apME-NLS::dsRed labeled nuclei in stage 17 LT muscles in control, in *1xDMef2-Gal4> 1xUAS-diaDN*::*GFP*, and in 2x*DMef2-Gal4> 2xUAS-diaDN*::*GFP* embryos **F.** Fusion index of Stage 17 lateral transverse (LT) muscles confirms the degree of fusion block in DiaDN embryos. In control embryos, 27.1±2.3 nuclei were counted in each hemisegment (n = 40 hemisegments). 1x*DMef2-Gal4>* 1x*UAS-diaDN*::*GFP* reduces the number of dsRed positive nuclei in each hemisegment to 22.5±2.2 (p<0.001), whereas 2x*DMef2-Gal4>* 2x*UAS-diaDN*::*GFP* further reduces the number to 15.5±2.7(p<0.001). Scale bar: 24μM.

The FH2 domain of mDia1, the mammalian homologue of *Drosophila* Dia, is required for its function in stress fiber generation in cultured cells **[62,63]**. A deletion of the first 21 amino acids of this domain was reported to act as a dominant negative protein, either by competing with endogenous mDia for F-actin binding or by binding to endogenous mDia to form non-functional dimers **[62,63]**. Since the key amino acids in this domain are identical between mDia1 and *Drosophila* Dia, we designed a *Drosophila* dominant negative Dia (DiaDN) modeled after this mouse construct (Fig 3A).

To confirm that the DiaDN construct affects Dia-based actin regulation, we examined filopodia in cultured cells and in the *Drosophila* epidermis. Previous work indicated that reduction of Dia leads to reduction of actin-based structures, such as filopodia, in cell culture and *in vivo* **[45,64]**. In S2R+ cells that express DiaDN the number of filopodia was greatly reduced compared to the neighboring control cells (5.7± 3.0 vs 17.5±5.8, n = 20, p<0.001) (Fig 3B), consistent with a reduction of endogenous Dia activity. This reduction in filopodia was rescued by overexpression of a full length Dia with DiaDN, revealing the specificity of the DN construct (S3C and S3D Fig). We also tested the efficiency of DiaDN *in vivo*, specifically by examining filopodia in leading edge (LE) cells during *Drosophila* dorsal closure. Similar to *dia* ^*5*^ maternal and zygotic mutant embryos **[45]**, filopodium number was reduced in embryos expressing DiaDN (Fig 3C–3Cii). Both these data sets are consistent with a reduction of Dia activity via our DiaDN construct. As additional test of our DiaDN construct, we examined another context in which Dia is known to play a role. *dia* ^*1*^ homozygous mutants are sterile due to defects in cytokinesis in the germline **[58]**. Expression of DiaDN in the male germline leads to reduced fertility due to fewer sperm (S3F and S3G Fig), consistent with a reduction in Dia activity in this context. Collectively, these data indicate the DiaDN construct reduces Dia activity.

We next examined the effects of DiaDN when expressed specifically in myoblasts. When one copy of DiaDN was expressed, we observed defects in muscle development, including myoblast fusion, in 50% of embryos (n = 20; Fig 3D). Other muscle differentiation processes also were disrupted, including muscle attachment and morphology (S3E Fig). These phenotypes were similar to those observed in *dia* mutant embryos (S2 Table; S3A and S3B Fig), reinforcing that the defects using DiaDN were due to Dia loss of function.

To increase the penetrance and expressivity of the fusion phenotype in embryos expressing DiaDN, we increased DiaDN expression levels in two ways: increasing genetic copy numbers of the mesoderm/muscle driver *DMef2-Gal4* driver and *UAS-DiaDN* and increasing the temperature at which we raised the embryos, since higher temperatures correlated with increased Gal4/UAS activity **[61,65]**. We generated a fusion index as described for *dia* mutants (Fig 3E and 3F; S3 Fig). Expression of one copy of *DiaDN* with one copy of the Gal4 driver (1X) resulted in a significant decrease in myoblast fusion (22.5±2.2 vs 27.1±2.3 LT nuclei/ hemisegment in control, p<0.001). Free myoblasts also were detected, in addition to detached muscles. Expression of two copies of *Dia DN* with two copies of the Gal4 driver (2X) resulted in a more severe fusion block (15.5±2.7 p<0.001) and more free myoblasts were observed. Under these conditions, some myotubes also failed to properly attach to tendon cells, and, as a result, formed myospheres (Fig 3D, arrowheads; S3E Fig). Specification and differentiation of both FCs and FCMs occurred normally under these conditions, as revealed by expression of MHC and apRed positive nuclei (Fig 3D and 3E) **[19,54]**. In combination with Dia’s localization at the fusion site, these data indicate that Dia activity is necessary for myoblast fusion.

**Fig 4:**
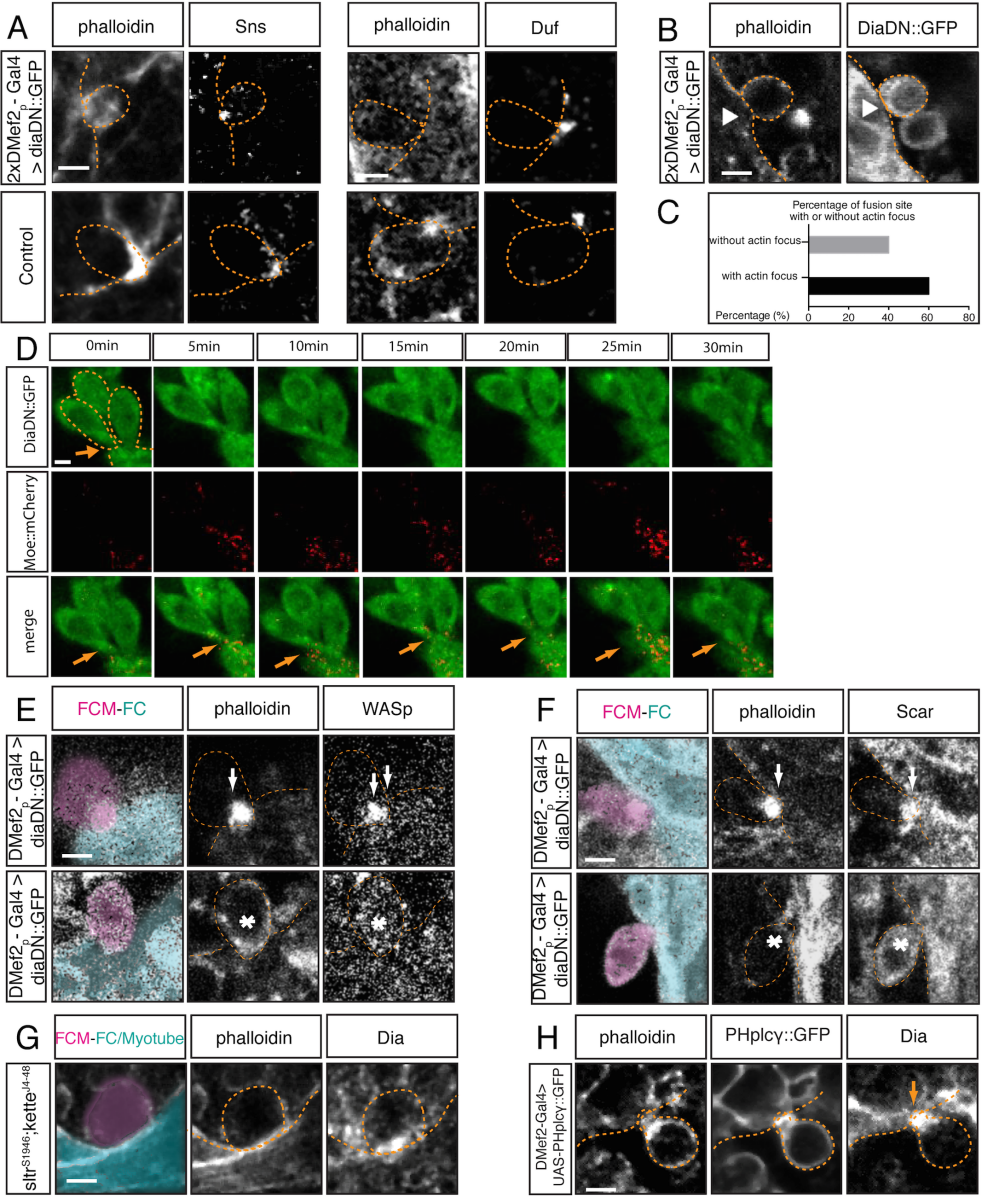
Dia regulates actin and Arp2/3 activity during myoblast fusion. **A.** In stage 15 embryos expressing 2x*UAS-diaDN*::*GFP*, myoblasts are stained with phalloidin and antibodies against DiaDN::GFP. Immunostaining for Sns and Duf was used to examine cell adhesion. Sns and Duf localize correctly at the fusion site, confirming adhesion between FCMs and FC/myotube. **B.** Stage 15 embryos expressing 2x*UAS-diaDN*::*GFP* with 2x*DMef2-Gal4*. Myoblasts are stained with phalloidin and antibodies against GFP. Arrowhead points to a myoblast adhering to the myotube but failing to generate a F-actin focus. **C.** Percentage of fusion sites with and without actin focus. Embryos expressing 2x *UAS-diaDN*::*GFP* were stained with phalloidin, and the formation of actin focus was quantified in these embryos. Actin focus forms in 60% of fusion sites and is absent in 40%. **D**. Time-lapse imaging of DiaDN fusion block. Three copies of *UAS-diaDN*::*GFP* and one copy of *UAS-moesin*::*mCherry* were driven by two copies of *Dmef2-Gal4*. F-actin dynamics were visualized by moesin::mCherry. Still images from the time-lapse sequence show 2–3 myoblasts adhered to a myotube (dashed lines), but unable to fuse. No F-actin focus was detected at the fusion site (arrow) (S1 Movie; S3I Fig). **E-F.** Comparison of SCAR and WASp localization in DiaDN-expressing FCMs. In embryos expressing high levels of DiaDN, immunostaining was used to examine the localization of SCAR and WASp. At a fusion site in which an actin focus forms (upper panels), SCAR and WASp both correctly localize to the actin focus (arrow; phalloidin). When actin focus formation is disrupted by DiaDN (lower panels), SCAR and WASp no longer accumulate at the fusion site (asterisk). **G.** Dia localization in *sltr* ^*s1946*^ *; kette* ^*J4-48*^ double mutant. In this double mutant, phalloidin was used to label F-actin, and Dia antibody to detect the localization of Dia. Fusion is blocked in double mutants, with no actin focus forming. Dia still accumulates at the fusion site. **H.** Dia localization in myoblasts expressing *UAS-PH* ^*plcγ*^::*GFP*. Stage 15 embryos expressing 2x*PH* ^*plcγ*^::*GFP* with 2x*DMef2-Gal4*. Myoblasts are labeled with antibodies against GFP. *PH* ^*plcγ*^ sequesters PI(4,5)P2, generating a small actin focus and blocking myoblast fusion**[54]**. In an FCM that adheres to a FC, immunostaining reveals that Dia accumulates at the fusion site. Scale bar: 2.5μM.

To determine which cellular step of fusion requires Dia activity, we next examined F-actin foci and myoblast morphology in embryos where *DiaDN*::*GFP* was expressed in myoblasts. We found that FCMs oriented towards the FC/Myotube, showing the characteristic teardrop shape **[8,19,56]**. FCMs also attached to FC/Myotubes. Localized expression of the recognition and adhesion receptors, Duf and Sns, at the fusion site confirmed that these FCMs were adhered to the FC/Myotube (Fig 4A). However, in 40% of the attached FCMs, an actin focus failed to form, consistent with the extent of fusion block found under these conditions (Fig 4B and 4C). Further confirmation of these data was obtained using time-lapse imaging of myoblast fusion (Fig 4D, S1 Movie, S3H Fig). Embryos expressing DiaDN::GFP in myoblasts showed movement of FCMs towards and attachment to FC/myotubes. A subset of these adhering FCMs showed no significant accumulation of actin that resembled the F-actin focus. The FCMs that failed to form a F-actin focus also failed to fuse to the FC/Myotube during the time-lapse sequence, consistent with reduction of Dia activity causing a fusion block. In agreement with Dia regulating actin at the fusion site, expression of DiaDN also significantly reduces actin focus size in the *blow* ^*1*^ mutant, which normally has an enlarged actin focus (S3I Fig). Together these data support the critical role of Dia for the formation of the F-actin focus as well as the importance of the F-actin focus during the fusion process.

Previous data **[21,54]** indicated that simultaneous loss of both Arp2/3 NPFs, SCAR and WASp, leads to a fusion block with no F-actin foci, due to the loss of Arp2/3 activity. To address whether the lack of the actin focus in the DiaDN expressing myoblasts was due just to reduced F-actin polymerization by Dia or whether Dia loss could also influence Arp2/3 activity, we examined the localization of the Arp2/3 regulators, SCAR and WASp, in myoblasts expressing the DiaDN construct (Fig 4E and 4F). We found that neither Arp2/3 regulator was present at the fusion site in those FCMs in which a focus failed to form (WASp: enrichment at 0% fusion site versus 100% in control. SCAR: enrichment at 20% fusion site vs 70% in control). These data suggest that Dia activity is required, through localization of SCAR and WASp, for Arp2/3 activity at the fusion site.

To further investigate whether Dia functions upstream of the Arp2/3 pathway, we examined Dia localization in *sltr* ^*s1946*^ *; kette* ^*J4-48*^ double mutants. In this double mutant, Arp2/3 is inactivated, due to the lack of activated SCAR and WASp, and no actin focus is observed at the fusion site **[54]**. Immunostaining of these mutants revealed that Dia accumulated at the fusion site (Fig 4G), suggesting that Dia accumulates at the fusion site prior to actin focus formation, and Dia’s localization is independent to Arp2/3 activity. Moreover, these data imply, that Dia expression, in the absence of Scar and Wasp activity (and by extension, absence of Arp2/3 activity), is not sufficient to build the F-actin focus.

As another test of our model, we examined Dia expression in embryos where PI(4,5)P2 signaling is abrogated. In other contexts **[66]**, PI(4,5)P2 signaling provides a localization cue for Dia. However, under conditions in which reduction of PI(4,5)P2 signaling leads to a myoblast fusion block **[54]**, Dia was still localized to the fusion site (Fig 4H). Hence, PI(4,5)P2 signaling appears not to be required for Dia localization during fusion. In addition, reduction in PI(4,5)P2 signaling at the fusion site leads to a reduction in actin focus size. PI(4,5)P2 signaling functions upstream of Arp2/3 activity and the reduced focus size correlated with reduced recruitment/maintenance/activity of Arp2/3 NPFs at the fusion site **[54]**. Localization of Dia at the fusion site in this background now provides, in part, a possible explanation for this smaller actin focus. Dia localization in this PI(4,5)P2 signaling mutant background would lead to low level recruitment/activity of Arp2/3 NPFs, subsequent Arp2/3 activity, and actin focus formation (albeit smaller). Nevertheless, Dia requires PI(4,5)P2 signaling to build an effective actin focus, capable of mediating myoblast fusion.

Taken together, we conclude that Dia is essential for myoblast fusion, and this function occurs after recognition and adhesion between FC and FCMs, but prior to Arp2/3-based actin polymerization. Importantly, Dia activity appears to be required for actin focus formation, both through its regulation of F-actin polymerization and the accumulation of Arp2/3 regulators at the fusion site.

### Constitutively Active Diaphanous Blocks Myoblast Fusion

**Fig 5:**
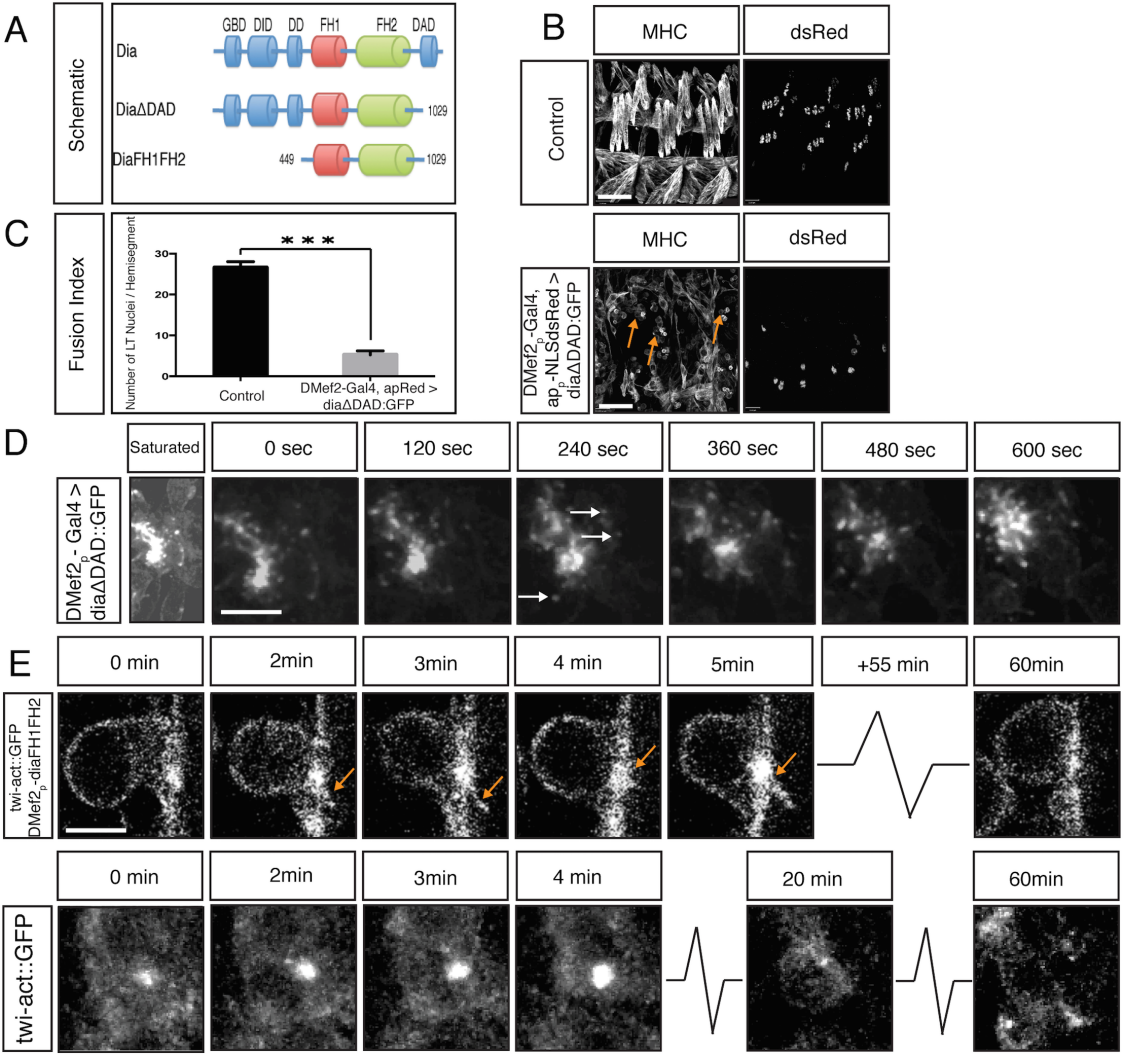
Constitutively active Dia blocks myoblast fusion. **A.** Schematic diagram of Dia domain structure and different constitutively active Dia deletion constructs used in this study. **B.** Whole mount lateral view of three hemisegments from stage 16 embryos showing the (MHC) labeled muscles and nuclei (apME-NLS::dsRed) of the lateral transverse (LT) muscles. Scale bar: 24μM. Expression of DiaCA blocks myoblast fusion as visualized by many free myoblasts (arrows). This fusion defect is not due to a failure in FC specification as witnessed by expression of apME-NLS::dsRed in nuclei. **C.** Fusion index confirms a total block in myoblast fusion: dsRed positive nuclei in LT muscles/ hemisegment were counted in control (26.6±1.5) and *DMef2-Gal4>UAS-diaΔDAD*::*GFP* (5.2±1.0) (n = 40 hemisegments/genotype) (p<0.001). **D.** Dynamics of DiaΔDAD::GFP expression in myoblasts. Still images from time-lapse of a stage 14 *DMef2-Gal4>UAS-diaΔDAD*::*GFP* embryo. Saturated image shows outline of cells and is used to localize myoblasts attempting to fuse. Filopodia-like protrusions undergo highly dynamic extension and retraction at areas of cell contact. DiaΔDAD::GFP localizes at the tip of those protrusions (arrows), and this signal moves as the filopodium extends and retracts (S3 Movie). Scale bar: 5μM **E.** Still images from time-lapse of stage 14 *twist-actin*::*GFP; DMef2-Gal4>UAS-diaFH1FH2* embryo. Images at 0 and 3 min show filopodia-like protrusions (arrows) emanating from the FCM, which adheres to the FC but is unable to fuse. The Actin::GFP signal is enriched at the fusion site during the entire time lapse sequence (1 hr). Compare to control FCM (*twist-actin*::*GFP*, lower panel), which fuses with FC in 30 minutes. Scale bar: 4μM

To gain further insight to Dia’s role in actin polymerization and in the localization of the Arp2/3 NPFs at the fusion site, we examined the effects of constitutively active Dia on myoblast fusion (Fig 5, S4 Fig, S5 Fig). Several well-characterized constitutively active Dia constructs (DiaCA) were employed, including DiaΔDAD, which has a deletion of the DAD domain, and FH1FH2, which consists of only Dia’s FH1 and FH2 domains (Fig 5A, S4A Fig) **[59]**. Expression of any of these DiaCA constructs in myoblasts blocked myoblast fusion in 100% of the embryos, as witnessed by the presence of unfused, free myoblasts (Fig 5B; S4B and S4C Fig). FCs were properly specified and myoblast recognition and adhesion, as measured by Sns and Duf localization, were unaffected (S5 Fig, S2 Movie); however, fusion did not occur (LT muscle fusion index: 5.2±1.0 vs 26.6±1.5, p<0.001/ hemisegment; Fig 5C; S4C Fig).

Examination of the localization of constitutively active Dia during myoblast fusion revealed that, as with endogenous Dia, all DiaCA constructs showed enrichment at the fusion site (Fig 5D, S4D Fig). Time lapse imaging showed, however, that DiaCA was associated with highly dynamic filopodia; for example, DiaΔDAD::GFP was found concentrated at the tip of each of the multiple filopodia at the fusion site (Fig 5D, arrows; S3 Movie). Under wild-type conditions or when Dia::GFP is overexpressed, such increased numbers of dynamic filopodia were not detected.

We next examined actin organization in myoblasts expressing DiaCA. GFP-tagged actin, which labels both G- and F-actin, was used to visualize actin (Fig 5E). We observed Actin::GFP accumulation at fusion sites, which were similar in size to that in control embryos (1.8±0.37μm vs 1.9±0.43μm, respectively; n = 50, p = 0.25). Multiple filopodia were also detected with Actin::GFP, extending from both the FCM and the FC/Myotube. The actin accumulation did not resolve during the 1h observation time, as fusion failed to occur. Together, these data indicate that DiaCA is recruited appropriately to the fusion site. There, DiaCA enhances actin polymerization, visualized as increased filopodia; however, this increase in actin polymerization appears not to be productive, as fusion progression is blocked.

### Constitutively Active Dia Alters Actin Dynamics and Organization at the Fusion Site

**Fig 6:**
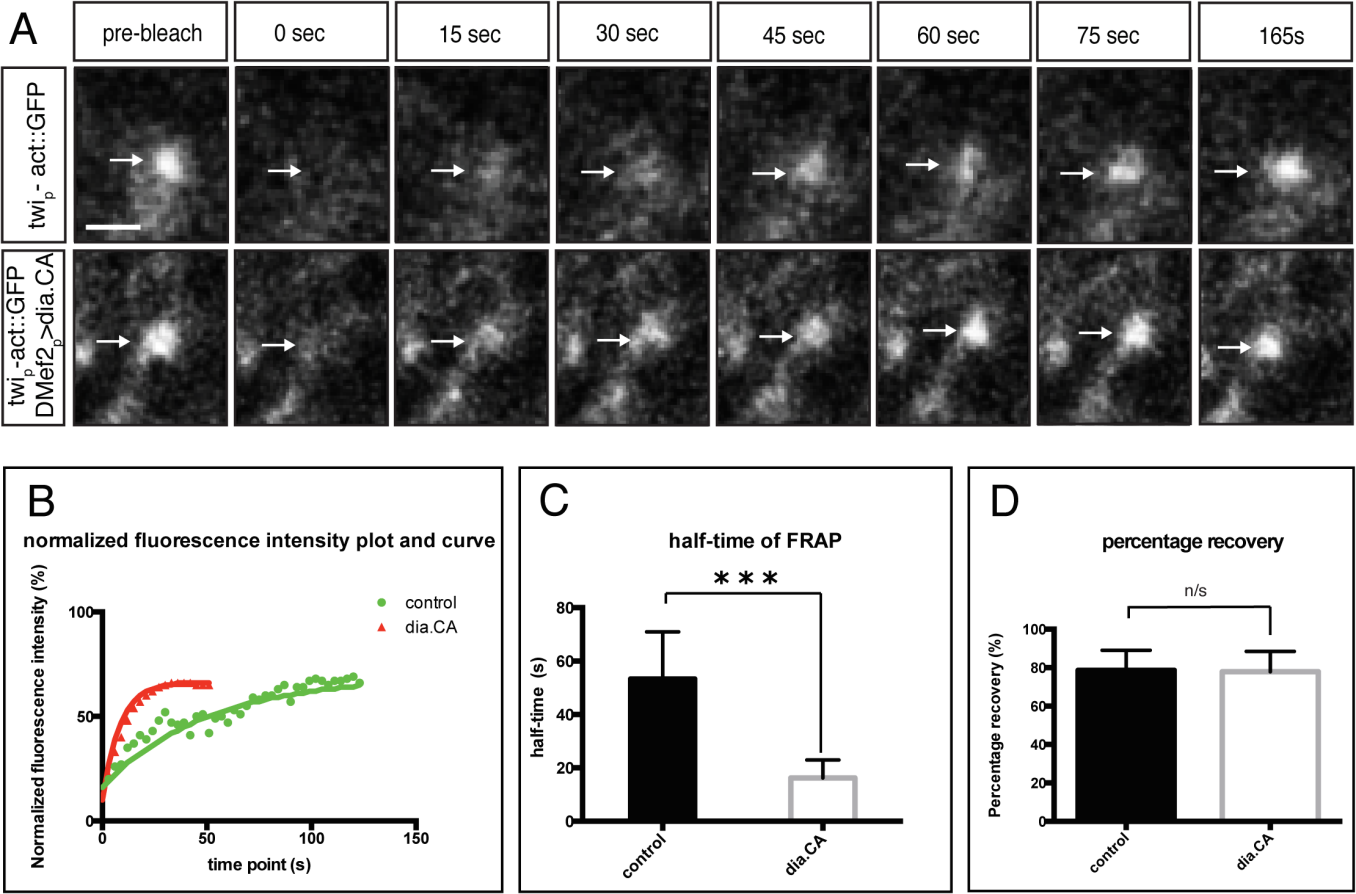
Constitutively active Diaphanous accelerates the actin exchange rate at the fusion site. Fluorescence recovery of Actin::GFP after photobleaching. Newly formed Actin::GFP foci in stage 14 embryos were photobleached (arrows) to approximately 30% of the original intensity. The recovery rate was recorded every 3s after photobleaching for a total of 165 sec (endpoint). **A.** Stills from time-lapse showing Actin::GFP recovery at an actin focus after photobleaching in wild-type and *DMef2-Gal4> UAS-diaFH1FH2* embryos. Scale bar: 2.5μM **B.** Comparison of representative recovery kinetics of Actin::GFP foci in control (green) and *DMef2-Gal4> UAS-diaFH1FH2* (red) myoblasts. **C.** Half time of fluorescent recovery in control (n = 8) and *DMef2-Gal4>UAS-diaFH1FH2* embryos (n = 10). The half time of fluorescence recovery in *DMef2-Gal4> UAS-diaFH1FH2* embryos (t_1/2_ = 17.7±6.7s) is significantly lower than control (t_1/2_ = 53.3±6.7s). **D.** Percentage of final recovery in control and *DMef2-Gal4>UAS-diaFH1FH2* embryos. Final recovery in *DMef2-Gal4> UAS-diaFH1FH2* embryos (77.9±10.6%) is similar to wild-type embryos (78.6±10.5%) (p>0.1).

The highly dynamic filopodia at the fusion site suggested that actin undergoes rapid remodeling in myoblasts expressing DiaCA. We employed fluorescence recovery after photobleaching (FRAP) to quantify the consequence of expressing DiaCA on actin dynamics at the fusion site. In control experiments, photobleaching of individual actin foci in wild-type embryos expressing Actin::GFP resulted in a rapid recovery of the fluorescent signal to pre-bleaching levels (Fig 6, S3 Table). Parallel experiments in myoblasts expressing both Actin::GFP and DiaCA revealed that DiaCA significantly enhanced the actin recovery rate relative to control (Fig 6A–6C): the half time of fluorescence recovery in embryos expressing DiaCA was significantly less than that in control (16.3±6.7s vs 53.3±17.7s, respectively, p<0.001). The percentage recovery for embryos expressing DiaCA, however, was similar to controls (Fig 6D). The rapid turnover rate of Actin::GFP upon DiaCA expression indicated that actin filaments undergo faster polymerization and depolymerization cycles than in control myoblasts. These data are consistent with the increase of rapidly extending filopodia observed in time lapse of myoblasts expressing constitutively active Dia.

**Fig 7:**
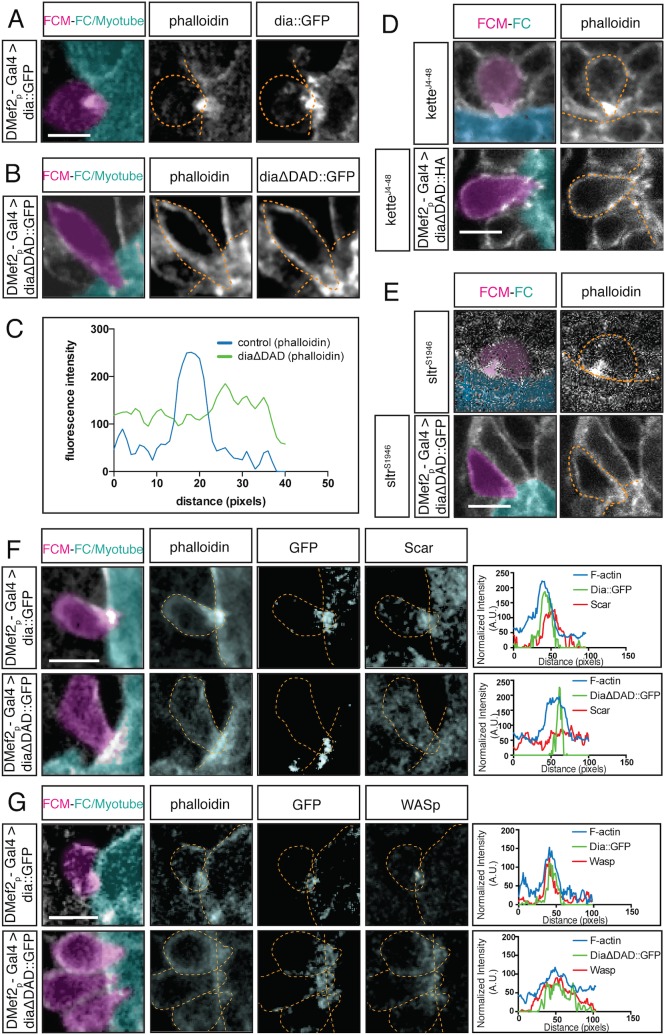
Constitutively active Dia alters the F-actin structure at the fusion site and regulates localization of Arp2/3 regulators. **A-B.** Stage 14 embryos stained for F-actin (phalloidin, white) and for Dia::GFP or DiaΔDAD::GFP (GFP antibody, white); FCM (magenta), FC/Myotube (turquoise). Scale bar: 5μM. **A.** Control *DMef2-Gal4>UAS-dia*::*GFP* myoblasts show colocalization of Dia::GFP and the F-actin focus at the fusion site. **B.** *DMef2-Gal4>UAS-diaΔDAD*::*GFP* myoblasts show that F-actin does not form a well-defined focus at the fusion site but appears diffuse. DiaΔDAD::GFP localizes to the plasma membrane and is enriched at cell contact sites. **C.** Fluorescent intensity curves confirm the distribution of F-actin in embryos expressing Dia::GFP and DiaΔDAD::GFP. **D-E.** Stage 15 embryos stained for F-actin (phalloidin) and DiaΔDAD::HA (antibodies against HA) showing FCM (magenta) and FC/Myotube (turquoise). Scale bar: 5μM. **D.** *DMef2-Gal4* driven expression of DiaΔDAD::HA in *kette* ^*J4-48*^ mutant background. The morphology of the F-actin focus at the fusion site appears similar to *DMef2-Gal4>UAS-diaΔDAD*::*GFP* embryos. While F-actin localizes at the cell cortex, it spreads out at the fusion site and does not make a concentrated focus. **E.** *DMef2-Gal4* driven expression of DiaΔDAD::GFP in *sltr* ^*s1946*^ mutant background. Similar to expression of DiaCA alone, the F-actin localizes at the cell cortex and spreads out at the fusion site. **F-G.** Stage 14 embryos stained for F-actin (phalloidin), Dia*ΔDAD*::GFP (GFP antibody) and SCAR or WASp. Scale bar: 5μM. **F.** SCAR localization in control and *DMef2-Gal4>UAS-diaΔDAD*::*GFP* embryos. In control embryos, SCAR accumulates at the fusion site, as confirmed by the fluorescent intensity curves. When expressing DiaΔDAD::GFP, SCAR loses its characteristic concentration at the fusion site and becomes found throughout the cytoplasm. The multiple peaks in the SCAR fluorescent intensity curve confirm SCAR’s change in localization. **G.** Localization of WASp in control and *DMef2-Gal4>UAS-diaΔDAD*::*GFP* embryo. In control embryos, WASp accumulates at the fusion site, as confirmed by the fluorescent intensity curves. When expressing DiaΔDAD::GFP, WASp displays a more diffused localization. Fluorescent intensity curves confirm the broader distribution of WASp signal in relation to the controls.

The previous experiments with Actin::GFP measured both G- and F-actin at the fusion site. To examine the organization and distribution of F-actin alone at the fusion site, we used phalloidin staining in fixed preparations. In myoblasts expressing DiaCA, the F-actin focus displayed a diffuse distribution rather than a compact spherical organization seen in wild-type FCMs (Fig 7A and 7B). This altered distribution was reflected in the fluorescence intensity curve: the peak of F-actin intensity curve in DiaCA myoblasts is broader in comparison to controls (Fig 7C). Together with the FRAP experiments, these data imply that DiaCA blocks myoblast fusion by altering actin dynamics and the actin focus organization at the fusion site.

### Constitutively Active Dia Leads to Mislocalization of Arp2/3 Regulators, SCAR and WASp

Our loss-of-function experiments indicate that Dia is required, not only for filamentous actin polymerization, but also for the localization of the Arp 2/3 NPFs, SCAR and WASp, at the fusion site. To better understand the link between Dia and the two Arp2/3 NPFs during fusion, we employed epistasis experiments, using the DiaCA and mutants in the NPF regulators (Fig 7). We first examined F-actin focus morphology in *kette* ^*J4-48*^ mutant embryos in which DiaCA is expressed in myoblasts. Kette regulates the stability and localization of SCAR during myoblast fusion. In *kette* mutants, myoblast fusion is blocked and F-actin foci are enlarged **[19]**. When expressing DiaCA in the *kette* ^*J4-48*^ background, F-actin did not form a dense focus, but rather, displayed diffuse localization, reminiscent of the F-actin distribution in DiaCA expressing myoblasts (Fig 7D). We also examined F-actin focus morphology *sltr* ^*S1946*^ mutant myoblasts; Sltr directly binds to and activates WASp. In *sltr* ^*S1946*^ mutants, myoblast fusion is blocked but the size of the F-actin focus is not changed **[19]**. When expressing DiaCA in the *sltr* ^*S1946*^ background, F-actin was diffuse at the fusion site and did not form a restricted focus, which again resembled F-actin distribution when DiaCA was expressed in myoblasts alone (Fig 7E). The distribution of F-actin in *kette* ^*-/-*^ and *sltr* ^*-/-*^ backgrounds when DiaCA was expressed suggested that Dia functions upstream of Arp2/3 in regulating F-actin assembly at the fusion site.

To confirm and build upon these data, we examined the localization of SCAR and WASp, the targets of Kette and Sltr activity in embryos expressing DiaCA. In control embryos, SCAR accumulated at the fusion site (Fig 7F). In embryos expressing DiaCA in myoblasts, SCAR was still present, but it was no longer enriched at, or restricted to, the fusion site: SCAR was found mislocalized throughout the FCM (Fig 7F). The mislocalization of SCAR was verified by fluorescence intensity curves: SCAR displayed several peaks in myoblasts expressing DiaCA, and only one overlapped with the F-actin. WASp also localized at the fusion site in control embryos (Fig 7G). In myoblasts expressing DiaCA, WASp, like SCAR, displayed a diffuse localization in the cytosol. Interestingly, when we evaluated the localization of WASp using fluorescence intensity curves, we found that WASp colocalized with the diffuse F-actin foci at the fusion site (Fig 7G). The mislocalization of SCAR and WASp suggests that, in myoblasts expressing DiaCA, Arp2/3 was activated over a larger area compared to control, therefore, could contribute to the diffuse localization of F-actin at the fusion site. The mislocalization of SCAR and WASp in both DiaDN and DiaCA expressing myoblasts, as well as the lack of an F-actin focus in myoblasts expressing DiaDN, indicates that Dia functions upstream of Arp2/3 and its regulators and that a particular level of Dia actin polymerization activity at the fusion site is required for optimal Arp2/3 activity and focus formation at the fusion site.

## Discussion

In this study, we provide the first evidence that the formin family member, Dia, is essential for *Drosophila* myoblast fusion. We show that Dia is expressed in all myoblasts and is recruited to the myoblast fusion site. The spatial and temporal distribution of Dia at the fusion site parallels that of the F-actin focus, which forms the core of an invasive podosome. This actin rich podosome is critical for FCM invasion of the FC/Myotube during fusion. In keeping with its expression pattern, Dia is essential for myoblast fusion progression: both loss and gain of Dia function lead to a fusion block. Under both conditions, the integrity of the F-actin focus and hence the invasive podosome is compromised; myoblasts expressing DiaDN fail to form the focus, whereas myoblasts expressing DiaCA have many filopodia and have a diffuse organization of F-actin, both of which contribute to a failure in invasive podosome formation and fusion. Dia activity is required after FC/Myotube and FCM recognition and adhesion but upstream of Arp2/3 activity. It is required, in parallel with PI(4,5)P2 signaling, to build a functional F-actin focus at the fusion site. Our experiments further indicate that Dia activity is critical for actin dynamics at the fusion site, which, in turn, regulate fusion progression. Moreover, the aberrant F-actin organization at the fusion site in both loss and gain of function is also due to altered localization of the Arp2/3 regulators, SCAR and WASp. Taken together, our data support a role for the formin Dia in a critical first step of actin polymerization at the fusion site, downstream of cell-cell recognition and adhesion, and link its activity to the formation of F-actin foci, required for myoblast fusion.

### Dia Is Required for Actin Polymerization at the Fusion Site

Actin remodeling is critical for myoblast fusion, but Arp2/3 was the only known actin polymerization factor that was shown to be necessary for myoblast fusion **[17,19]**. We now show that the formin Dia is also required during myoblast fusion. Whereas Arp2/3 preferably binds to pre-existing actin filaments and generates uncapped F-actin, formins nucleate F-actin both *de novo* and from the barbed ends of pre-existing actin filaments. Thus, Dia can generate actin filaments *de novo*, which Arp2/3 can bind or elongate **[67,68]**.

We also show that the level of Dia activity is critical for myoblast fusion. Too much actin polymerization leads to too many filopodia and absence of an invasive podosome with its characteristic F-actin core. Too little polymerization leads no actin focus and no podosome formation. Our FRAP data with DiaCA also hint at whether a limited pool of actin is available for the actin polymerization factors during myoblast fusion. Despite the high rates of actin turnover with expression of DiaCA, the final fluorescence levels of actin returns to the same value as in controls. Additional actin monomers are not recruited to the site, even with high levels of polymerization activity. Interestingly, the rate of actin turnover has also been measured in mutants that affect Arp2/3 activity: specifically, mutations in *blow*, which regulates the Arp2/3 NPF WASp, show lower rates of actin exchange than in controls, due to a reduced exchange rate for WASp on the barbed ends of actin at the fusion site **[30]**. Together these data suggest future experiments aimed at examination of whether rates of actin polymerization regulated by both Dia and Arp2/3 are optimized for the available actin pool and tightly controlled for myoblast fusion to properly occur.

### Dia and Arp2/3 Activities Are Linked during Myoblast Fusion

Both cooperative and antagonistic functions between Dia and Arp2/3 have been reported **[69,70]**. Here we demonstrate that the coordinated and cooperative activities of these two actin polymerization factors leads to the formation of the F-actin focus. With the exception of *sltr*/*Dwip*/*vrp* mutants that form a focus of wild-type size, single mutants in the Arp2/3 NPF pathways, WASp and SCAR, lead to enlarged foci; however, double mutants in WASp and SCAR pathways do not form foci **[54]**. This is the same phenotype that we have seen in myoblasts expressing the DiaDN. Our data support Dia activity being upstream of WASp and SCAR activation of Arp2/3 at the fusion site. This suggests that, at the fusion site, Dia initially provides the necessary context upon which Arp2/3 can act and not vice versa, as has been suggested in other contexts in which linear actin filaments emerge from Arp2/3 based structures **[30]**. Nevertheless, both sets of actin regulators are necessary for F-actin focus formation that provides the core of the invasive podosome. Neither Dia nor Arp2/3 alone are sufficient.

The interplay between Dia and Arp2/3 at the fusion site is also reflected by our localization studies. Too little or too much Dia activity resulted in improper localization and, by extension, improper activity of Arp2/3 NPFs. How could Dia regulate this localization? One possibility is that Dia indirectly regulates Arp2/3 localization. Dia could nucleate linear actin filaments, which then would provide the necessary substrate for recruitment, maintenance and /or activation of Arp2/3 and its regulators, such as the WASp-WIP complex **[30]**. Another possibility is that Dia, through its interactions with members of the SCAR/WAVE complex such as Abi, may directly localize and/or maintain the localization of Arp2/3 regulators, which are then activated at the fusion site. Abi has been reported to bind directly with Dia *in vitro*, and this interaction is required for the formation and stabilization of cell-cell junctions **[34]**. Dia likely changes the localization and integrity of the SCAR/WAVE complex by competitively binding to the N-terminal part of Abi, dissociating Kette/Nap1 from the complex, and thus changing the stability and localization of SCAR/WAVE. It has also been established that the recognition and adhesion receptor, Sns, is capable of recruiting the Arp2/3 NPFs, such as WASp, to the fusion site **[30]**. While Sns is still clustered at the fusion site in DiaDN and DiaCA, its recruitment activity appears not sufficient for focus formation capable of supporting an invasive podosome.

We have shown that localization of Arp2/3 NPFs is affected in Dia loss and gain of function. In addition to this spatial control, another important way of controlling Arp2/3 activity is through activation of the NPFs via small GTPases. SCAR is activated through Rac-dependent dissociation from SCAR inhibitory complex **[19,26,27]**. WASp is activated by binding to Cdc42, which releases it from auto-inhibited state **[17,18,19,20]**. In this study, we did not examine the localization of these activated GTPases. However, previous work has shown that PI(4,5)P2 signaling is required for proper localization of activated Rac at the fusion site **[54]**. How the localization and activity of small GTPases at the fusion site contribute to the spatial and temporal interplay between Dia and Arp2/3 regulation of actin polymerization requires further investigation.

It remains unresolved how Dia itself is recruited to the fusion site. Our data suggest that the recognition and adhesion receptors Duf and Sns would be involved either directly or indirectly in recruiting Dia to the fusion site, as embryos that fail to express either of these adhesion receptors fail to recruit Dia to the fusion site. In addition, recent data from *Drosophila* epithelial tubes **[66]** indicate that PI(4,5)P2 serves as a localization cue for Dia. Previous work in our lab has shown that PI(4,5)P2 accumulates at the fusion site after FC-FCM recognition and adhesion; sequestering of PI(4,5)P2 results in a significant fusion block **[54]**. We thus tested whether PI(4,5)P2 regulates Dia localization at the fusion site. We find that Dia is recruited to the fusion site in the PI(4,5)P2 sequestered myoblasts, suggesting that, in this context, PI(4,5)P2 signaling is not required for Dia localization. These data provide possible explanations for why in PI(4,5)P2 sequestering embryos, smaller actin foci are detected: the localized Dia may be sufficient to recruit low levels of Arp2/3 and its NPFs, which, upon activation, lead to the formation of small F-actin foci. Nevertheless, in the absence of PI(4,5)P2 signaling, Dia that is recruited to the fusion site is not sufficient to produce functional actin focus, capable of directing a fusion event. Recent work **[71]** also indicates that charged residues in the N- and C-termini of mDia1 are sufficient both for mDia’s clustering of PI(4,5)P2 and its own membrane anchorage. This interaction between mDia1 and PI(4,5)P2, in turn, regulates mDia1 activity. Whether such a mechanism is in play at the myoblast fusion site needs to be further investigated.

### A Model for Interactions between Actin Polymerization Factors during Myoblast Fusion

**Fig 8:**
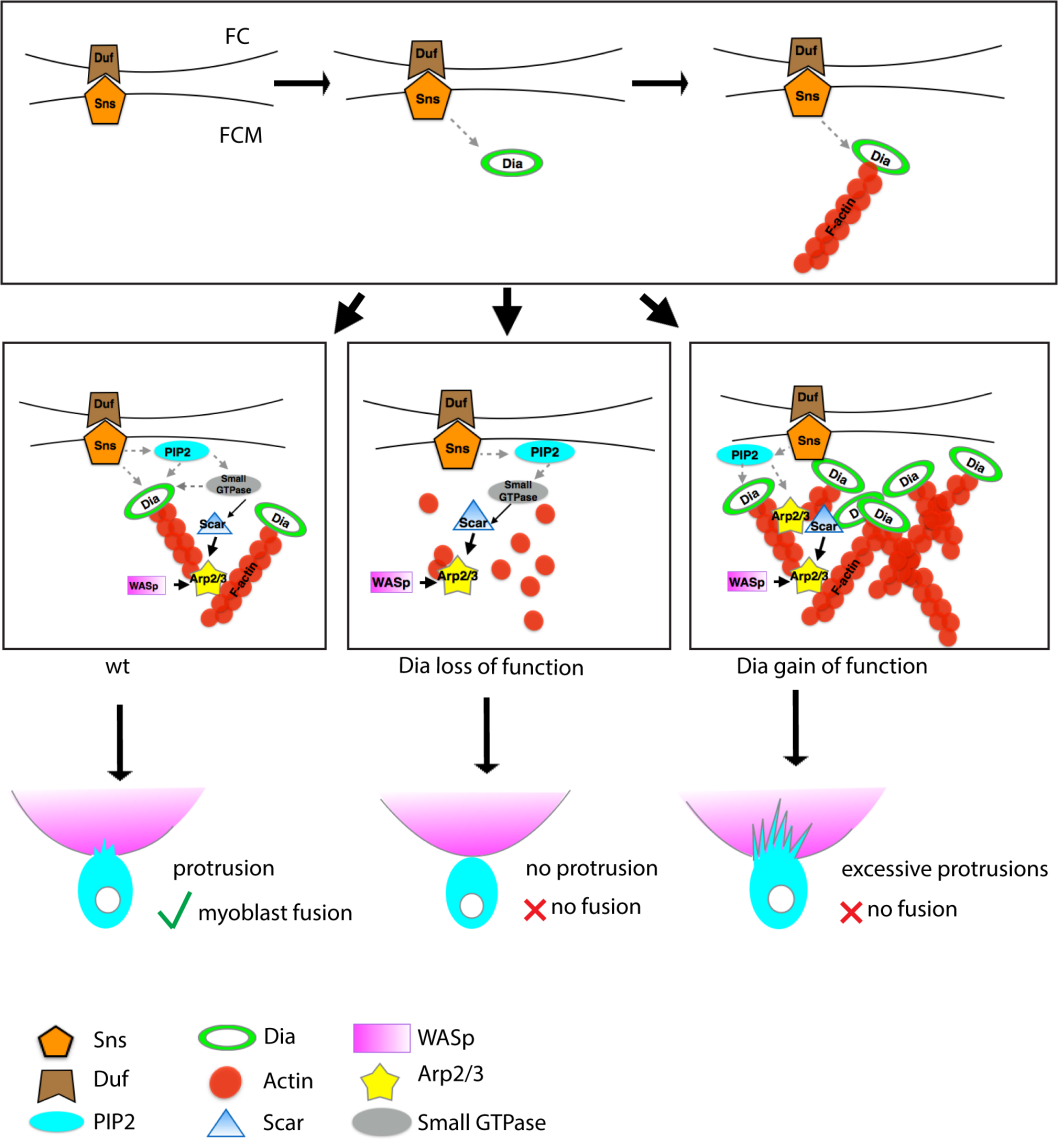
Model: Dia and Arp2/3 function together to regulate myoblast fusion. During myoblast fusion, the transmembrane molecules (e.g. Sns and Duf) mediate recognition and adhesion between the FCM and the FC/myotube. After cell adhesion, Dia is recruited to the fusion site, where it collaborates with PI(4,5)P2 signaling in making a functional F-actin focus, How PI(4,5)P2 signaling coordinates with Dia is unclear, but it may possibly be through the recruitment of small GTPases that activate Dia. Active Dia, in turn, nucleates F-actin particularly on the FCM side, which serves as a substrate for Arp2/3. Coordinated Dia and Arp2/3 activities allow the actin network to consolidate into invasive podosome-like structures. Loss of filamentous actin and reduction in Arp2/3 NPFs recruitment/maintenance due to Dia loss results in Arp2/3 being unable to nucleate enough actin filaments to build an invasive podosome-like structure. Hence no fusion can occur. In Dia gain of function embryos, Dia builds excessive actin filaments. Arp2/3 NPFs fail to localize properly, leading to an alteration in the distribution of activated Arp2/3. Since the actin network fails to consolidate into invasive podosome-like structures, no myoblast fusion can occur.

We propose a working model for the interplay between the actin regulators during myoblast fusion (Fig 8). Dia is recruited to the fusion site upon engagement of the recognition and adhesion receptors by a yet-to-be determined mechanism. We propose that PI(4,5)P2 signaling at the fusion site regulates the localization and activation of downstream targets such as Rho-family of small GTPases. These small GTPases lead to the activation of Dia. Activated Dia, in turn, polymerizes linear actin filaments and, in combination with the recognition and adhesion receptors and PI(4,5)P2, recruits the Arp2/3 NPFs, SCAR and WASp. Activation of these Arp2/3 NPFs at the fusion site would be accomplished by small GTPases such as Rac. These, in turn, would activate Arp2/3, leading to branched actin and formation of the F-actin focus and the invasive podosome. Whether the Arp2/3 NPFs such as SCAR/WAVE would negatively regulate Dia to downregulate linear actin polymerization, as suggested for mDia2 in cell culture **[35]**, or whether Dia competes with WASp for barbed end binding remains to be investigated**[72]**. However, these mechanisms would underscore a switch from linear F-actin filopodium formation to the linear and branched F-actin invasive podosome–like structure that is necessary for fusion.

### Invasive Podosomes in Development and Disease

The actin focus formed at the fusion site is an F-actin rich, invasive podosome-like structure that has been suggested to provide a mechanical force for FCMs to invade the FC/Myotube **[30]**. Similar invasive actin structures named invadosomes have been seen in different cell types, such as podosomes in macrophages and invadopodia in cancer cells **[73]**. Arp2/3 is known to play a key role in invadosome formation, and recent studies have revealed the involvement of formins in developing invadosomes **[38,39]**. Our data indicate that specific temporal and spatial interactions between the formin Dia and Arp2/3 are required for the actin focus and invasive podosome formation. Our data thus provide new mechanistic insights for the interplay of Arp2/3 and Formins during invadosome formation in these contexts.

## Methods

### Fly Stocks

The following stocks were used: *Oregon R* (control, wildtype), *UAS-diaFH1FH2* (Bloomington #27616), *twist-actin*::*GFP* **[19,74]**, *apME-NLS*::*dsRed* **[19]**, *UAS-dia*::*GFP*, *UAS-DiaΔDAD*::*GFP*; *UAS-diaFH1FH2*::*GFP*, *UAS- DiaΔDAD*::*HA*, *UAS-DiaDDFH1FH2*::*GFP* (M. Peifer) **[45]**, *duf5*.*1-Gal4* (5.1kb enhancer region of *duf* fused with Gal4, sequence information based on **[14,75]**), *blow* ^[^ ^1^ ^]^ **[56]**, *sns* ^[XB3]^ **[13]**, *mbc* ^*[c1]*^ **[55]**, *kette* ^*[J4-48]*^ **[76]**, *loner* ^*[T1032]*^ **[57]**, *sltr* ^*[S1946]*^ **[20]**, *Rac1* ^*[J11]*^, *Rac2* ^*[Δ]*^, *Mtl* ^*[Δ]*^ **[77]**, *wsp* ^*[3D3-35]*^ **[78]**. Stocks were balanced over *CyO*, *dfd-GMR-YFP* or *TM3*, *dfd-GMR-YFP* **[79]**, and identified by GFP staining. *UAS-moesin*::*mCherry* **[53]**, *DMef2*-*Gal4* **[80]**, *dia* ^5^ **[40]**, *dia* ^*2*^ **[58,59]**. *UAS*-*DiaDN*::*GFP* (this study), *UAS-PH* ^*plcγ*^::*GFP* **[81]**, *net-Gal4;nos-Gal4* **[82,83]**. The GAL4-UAS system **[61]** was used for expression studies. Embryos were staged according to **[84]**.

### Immunohistochemistry

Embryos were collected, fixed in 4% PFA, and stained according to standard lab protocols **[19]**. Antibodies were used at the following concentrations: α-Dia (1:500) (S. Wasserman), α-Duf (1:200) (K.-F. Fischbach), α-Sns (1:250) (S. Abmayr), α-WASp (1:500) (E. Schejter), α-SCAR(1:100) (J Zallen) **[85]**, α-Blow (1:100) (E. Chen), α-GFP (1:500)(Invitrogen A11120), α-dsRed (1: 500) (Clontech 32392), Alexa Fluor 647-phalloidin (1:100) (Invitrogen A22287). For secondary antibodies, Alexa Fluor 488-, Alexa Fluor 555-, and Alexa Fluor 647-conjugated fluorescent secondary antibodies at 1:200 dilution (Invitrogen) were used. Fluorescent images were acquired on a Leica SP5 laser scanning confocal microscope equipped with a 63X 1.4 NA HCX PL Apochromat oil objective and LAS AF 2.2 software. Maximum intensity projections of confocal Z-stacks were rendered using Volocity visualization software (Improvision). Cell outlines of somatic myoblasts and myotubes were determined in saturated images, and cells were false colored using Adobe Photoshop CS4 (S1 Fig).

### Fusion Index Quantification

Nuclei in the four LT muscles were specifically labeled by expressing dsRed fused to a nuclear localization signal under the control of the *apterous* mesodermal enhancer (apRed) **[19,86]**. Fusion index was quantified by counting the number of dsRed positive nuclei in each hemisegment in stage 17 embryos. 10–40 hemisegments were analyzed.

### Line Scan for Measuring Fluorescence Enrichment at Actin Foci

Line scans were done in ImageJ **[87]**. The focal plane was selected when the cross sectional area of F-actin focus was its largest **[19]**. A line of predetermined length was drawn across the F-actin focus (Fig 1), or along the FCM cell outline that was adhered to the myotube (all other Figures). Grey values were measured along the line. The relative intensity was calculated at each point by y_relative_ = (y-y_min_)/(y_max_-y_min_). Five to ten F-actin foci were measured for each genotype. The average relative intensity and standard deviation of mean at each point were calculated from those samples. Fluorescence intensity along the line was similarly measured for each of the different channels. The different curves were aligned according to the location of the line. For control, a line of the same length was dropped outside the cell. Signal intensity was measured, normalized, and averaged in the same way as described above. The pipeline for our line scan analysis is shown in S2 Fig.

### Spermatogenesis

Male adult flies carrying *net-Gal4;nos-Gal4>UAS-diaDN*::*GFP* or *net-Gal4;nos-Gal4>w* (ctrl) **[82,83]** were crossed to w female in a 1:2 ratio. Mated females were allowed to lay eggs for four days in 29°C. The number of larvae were counted, and this number was used to determine the number of larvae produced per male per day. Testis and seminal vesicles were dissected (n = 5), fixed in 4% PFA, and stained with DAPI **[88]**. Sperm numbers were quantified using images taken on confocal microscope.

### Fluorescence Recovery after Photobleaching

Actin::GFP was used to label F-actin foci at fusion site. FRAP experiments were performed on a Zeiss LSM710, with 40x oil objective. 488nm laser was set at 14%. Pinhole was set at AU 1.0. The region of interest (ROI) was identified and selected manually, and two scans were taken before bleaching. 100% 488nm and 458nm laser were used to bleach ROI to approximately 30% of the original intensity. After photobleaching, images were acquired every 3s. For data analysis, the size of ROI was fixed for each movie, but the location was adjusted manually for each frame, as the F-actin focus shifts over time. Fluorescence intensity was measured in ImageJ and was normalized to background. Half time was determined by y = y_min_+(y_max_-y_min_)(1-e^-kt^), and kinetic curves were plotted according to the calculation **[30]**.

### Molecular Cloning

Dominant negative Dia (DiaDN) was designed according to **[63]**. In brief, a dominant negative mDia construct was developed by creating a 21 amino acid deletion (spanning aa 750–779) in the FH2 domain within aa 567–1182, which contains the FH1 and FH2 domains (Fig 3). Alignment of mDia1 with *Drosophila* Dia identified a conserved 497–1029 amino acid sequence containing the FH1 and FH2 domains. The orthologous 21 aa deletion spanning aa 599–619 in FH2 was created; this construct, containing the FH1 domain and part of the FH2 domain, served as our dominant negative Dia (DiaDN) in *Drosophila*. We cloned DiaDN into pUAST vector with a C-terminal GFP or mCherry tag using two pairs of primers: 5’CACCGAATTCATGGGTGTGGCGGCTCCGTC3’ and 5’AAAAGATCTGCCATGAGGCAGAACGGG3’, 5’CACCGGATCCGTCCCGGCCAAAATGTCC3’ and 5'AAAGGATCCCATCACGCCTTCCTGCG 3'. The DiaDN constructs were validated by sequencing and tested in S2 Cells and *in vivo*.

### Time-lapse Imaging

Embryos were raised at 25°C, collected, dechorinated and mounted on a Teflon membrane in Halocarbon oil 700 (Halocarbon Products Corp., Series 700, 9002-83-9). Images were acquired every 30 or 60 sec as indicated, single z = 0.5μm 18-20mm total on an upright Leica SP5 laser scanning confocal microscope equipped with a 63X 1.4 NA HCX PL Apochromat oil objective and LAS AF 2.2 software. Maximum intensity projections of confocal Z-stacks were rendered using Volocity visualization software (Improvision).

### Cell Culture

S2R+ cells were grown in Schneider’s medium. Transfection was done in Grace’s medium with Cellfectin II reagent (Invitrogen 10362–100). 0.5μg of UAS-DiaDN::GFP and 0.5μg of actin-Gal4 were co-transfected in S2R+ cells. Cells were incubated in 25°C for 24h, and fixed in 4% PFA. Phalloidin was used to label F-actin in S2R+ cells. For the rescue experiment, 0.5μg of UAS-dia::GFP, 0.5μg UAS-diaDN::mCherry and 2.5μg of ubi-Gal4 were co-transfected in S2R+ cells. Cells were incubated in 25°C for 24h, and fixed in 4% PFA. Phalloidin was used to label F-actin in S2R+ cells, filopodia number was counted in 5–20 cells.

### Statistical Analysis

Statistic tests were done in Microsoft Excel and GraphPad Prism. The Student’s t-test was used to compare the mean of two groups. One-way ANOVA test was used to compare mean of three or more groups. The difference between two groups was considered significant when p-value<0.1.

## Supporting Information

S1 Table Quantification of Dia enrichment at the fusion site in control and fusion mutants. (DOCX)

S2 Table Quantification of muscle phenotypes in *dia* mutant and *dia* knockdown. (DOCX)

S3 Table FRAP summary: Actin::GFP recovery rate and percentage of recovery in control embryos and constitutively active Diaphanous embryos. (DOCX)

S1 Movie Time-lapse imaging of an embryo expressing DiaDN::GFP in myoblasts: no actin focus and no fusion. Time-lapse imaging of a stage 15 embryo with the genotype: 3X *UAS-diaDN*::*GFP; 2X DMef2-Gal4 and UAS-moesin*::*mCherry*. Myoblasts / myotubes are labeled by DiaDN::GFP (green), F-actin dynamics are visualized with moesin::mCherry (red). Arrow shows an attachment site between myoblasts and a myotube. No actin accumulation is observed at this site. Arrowhead shows a detached myoblast with actin accumulation.
(MOV)

S2 Movie 3D structure of fusion site in embryos expressing DiaΔDAD::GFP. Sns localization in stage 15 *DMef2-Gal4>UAS-diaΔDAD*::*GFP* embryo. Immunofluorescence shows Sns strongly accumulates at the fusion site.
(MOV)

S3 Movie Dynamics of filopodium generated by DiaCA at the fusion site. Time-lapse movie of a stage 14 *DMef2-Gal4>UAS-diaΔDAD*::*GFP* embryo. Highly dynamic protrusions are seen at the fusion site (arrow), with DiaΔDAD::GFP localized at the tip of each protrusion.
(MOV)

S1 Fig Dia expression in fusing myoblasts. **A.** Projection of three hemisegments of a stage 14 *twist-actin*::*GFP* embryo stained for GFP which reveals the myoblasts in each hemisegments as fusion occurs. **Ai.** Three hemisegments of a stage 16 control embryo stained for MHC. This image reveals the muscle pattern that results from myoblast fusion. Scale bar: 10μM **B.** Higher magnification image showing single scan of the boxed area: one hemisegment from a stage 14 *twist-actin*::*GFP* embryo stained for F-actin (phalloidin) and antibodies against Dia and GFP. The F-actin focus at the fusion site was visualized both by GFP antibody (green; *twist-actin*::*GFP*) and phalloidin staining (white). Dia (red) is present in muscles and is enriched at fusion sites (arrows). Scale bar: 10μM. **C.** Cell outline determination used in all Figures: we determine the FCM and myotube cell outlines manually by adjusting brightness and changing focal planes.
(TIF)

S2 Fig Pipeline of measuring fluorescent intensity is measured. For all Figures except Fig 1: a line was drawn along cell cortex, with the center of the line localized at the actin focus or cell contact site. Fluorescent intensities were measured along the line and normalized using the equation shown in Figure. After the desired number of samples was measured, average relative intensities and standard deviations at each point were calculated and plotted.
(TIF)

S3 Fig Muscle phenotypes in *dia loss of function* embryos. **A.** Stage 16 *dia* ^*2*^ and *dia* ^*5*^ homozygous embryos were stained with MHC to visualize muscle pattern. GFP antibody was used to identify balancer. *dia* ^*2*^ is an amorphic allele and *dia* ^*5*^ is a hypomorphic allele; both result in Dia loss of function. Homozygous *dia* mutants display a range of muscle defects, including muscle detachment (arrowhead), missing muscles (asterisk), free myoblasts (arrow), and muscle shape changes. Scale bar: 40μM. **B.** Fusion index of *dia* ^*2*^ and *dia* ^*5*^ homozygous mutants. *apMe-NLS*::*dsRed* was expressed in LT muscles to label nuclei, and fusion index was assessed in stage 17 embryos. Fusion was impaired in *dia* ^*2*^ and *dia* ^*5*^ homozygous mutant embryos (*dia* ^*2*^: 14.1±1.3, n = 21, *dia* ^*5*^: 21.5±1.3 n = 20 vs control: 27.8±0.3 n = 12, p<0.001). **C.** Expression of Dia::GFP rescued filopodia reduction by DiaDN. S2R+ cells that were co-transfected with Dia::GFP (green), and/or DiaDN::mCherry (red) (n = 7). Expression of DiaDN::mCherry results in a significant reduction of filopodia (phalloidin, grey in single channel). By contrast, expression of Dia::GFP resulted in increased cell spreading and increased numbers of filopodia (phalloidin, grey) compared to control. Expression of both constructs simultaneously results in more filopodia-like, protrusive structures relative to cells that are transfected with DiaDN::mCherry alone (n = 5). Scale bar: 10μM. **D.** Filopodia numbers were quantified in cells expressing DiaDN::mCherry, DiaDN::mCherry+Dia::GFP, Dia::GFP and mock treated control. **E**. Two copies of *DMef2-Gal4* driving two copies of *UAS-diaDN*::*GFP* in myoblasts at 29°C. Antibodies to GFP were used to visualize the muscle cells. Embryonic muscle defects were found, including free myoblasts (arrow) and missing muscles (asterisk) at Stage 16 and severe muscle detachment (arrowhead) at Stage 17. Scale bar: 20μM and 40μM from left to right. **F-G.** In male flies which carry *net-Gal4;nos-Gal4>UAS-diaDN*::*GFP*, male fertility (**F**) and sperm number (**G**) were quantified and compared to control. **H.** Time-lapse imaging showing a fusion site in embryos expressing DiaDN::GFP. Three copies of *UAS-diaDN*::*GFP* and one copy of *UAS-moesin*::*mCherry* were driven by two copies of *Dmef2-Gal4*. F-actin dynamics were visualized by moesin::mCherry. As DiaDN does not block myoblast fusion completely, fusion events can be observed. Still images from the time-lapse sequence show a myoblast fusing to a myotube, with a normal F-actin accumulation at the fusion site. Scale bar: 5μm **I.** Quantification of actin focus size of *blow* ^*1*^ embryo and *blow* ^*1*^ *;DMef2-Gal4*,*UAS-diaDN*::*GFP* embryos. The actin focus size is significantly reduced in *blow* ^*1*^ *;DMef2-Gal4*,*UAS-diaDN*::*GFP* embryos (n = 18) compared to that in the *blow* ^*1*^ embryo (n = 18, P<0.01).
(TIF)

S4 Fig Embryonic phenotypes found in embryos expressing different constitutively active Dia constructs. **A.** Schematic diagram of Dia domain structure and the different constitutively active Dia (DiaCA) deletion constructs (DiaΔDAD, diaDDFH1FH2, DiaFH1FH2) used in this study. **B-C.** Muscle pattern in a single hemisegment and Fusion index in embryos expressing the different DiaCA constructs. *UAS-diaCA* was expressed in muscles using the *DMef2-Gal4* driver. MHC staining shows that myoblast fusion was blocked by DiaCA (high magnification of the LT Muscle area). To confirm this observation, apMe-NLS::dsRed was expressed in LT muscles to measure the fusion index. Compared with controls, all *diaCA* constructs significantly reduce myoblast fusion (graph in B. p<0.001). **D.** F-actin structure and DiaCA localization when expressing different *diaCA* constructs. F-actin was labeled with phalloidin. DiaCA localizations were visualized with GFP immunofluorescence staining. Instead of forming a defined actin focus at the fusion site, F-actin displayed a diffused localization in myoblasts expressing DiaCA. DiaDDFH1FH2 and DiaFH1FH2 both localize in the cytoplasm, while DiaΔDAD localizes primarily at the membrane. Intensity plot shows the colocalization of DiaCA (green) and actin structure (red) at the fusion site. Scale bars in **B-D**: 10μM.
(TIF)

S5 Fig Recognition and adhesion are not affected by constitutively active Dia. **A.** Sns localization in control (DMef2-Gal4>UAS-dia::GFP) and DMef2-Gal4>UAS-diaΔDAD::GFP embryos. Stage 14 embryos stained with phalloidin and antibodies against GFP and Sns. In control embryos, Sns is localized at the fusion site on the FCM side. Constitutively active Dia does not change Sns localization at the fusion site. Fluorescent intensity curves confirm that the Sns peak colocalizes with F-actin peak in both control and DMef2-Gal4>UAS-diaΔDAD::GFP embryos. **B.** Duf localization in control and DMef2-Gal4>UAS-diaΔDAD::GFP embryos. Stage 14 embryos stained with phalloidin and antibodies against GFP and Duf. In control embryos, Duf is localized at the fusion site on the FC/myotube side. Similar to Sns, Duf localization is not changed by DiaΔDAD::GFP. Fluorescent intensity curves confirm that the Duf peak colocalizes with the F-actin peak for both control and DMef2-Gal4/UAS-diaΔDAD::GFP embryos. The peak of Duf is broader and less defined in DMef2-Gal4>UAS-diaΔDAD::GFP embryos. **C-D.** Projection image of myoblasts expressing the constitutively active Dia construct DiaΔDAD. Actin (phalloidin, white); Dia (GFP antibody, green), Sns or Duf (antibody, Red). Dashed lines indicate FCMS adhered to the FC. Compared to the control embryo, the accumulation of Sns and Duf appears stronger due to an increased number of unfused and adhered myoblasts. Scale bar: 2.5μM.
(TIF)
