# Supplementary material for: The Formin Diaphanous Regulates Myoblast Fusion through Actin Polymerization and Arp2/3 Regulation
Source: PLoS Genet. 2015 Aug 21;11(8):e1005381. doi: 10.1371/journal.pgen.1005381 (PMC4546610; doi:10.1371/journal.pgen.1005381)
Supplement: S1 Table — (DOCX) [file pgen.1005381.s001.docx]

Table: Diaphanous enrichment at the actin focus

|  | Number of embryos examined | Number of foci examined | Number of Dia Positive foci | Percentage of Dia positive foci |
| --- | --- | --- | --- | --- |
| *twi-actin::GFP* | 7 | 40 | 40 | 100% |
| *sns^XB3^/Cyo* | 1 | 5 | 5 | 100% |
| *sns^XB3^* | 4 | 0^[1]^ | 0 | 0 |
| *rac1^J11^,rac2^Δ^,mtl^Δ^/TM3* | 1 | 5 | 5 | 100% |
| *rac1^J11^,rac2^Δ^,mtl^Δ^* | 5 | 25 | 24 | 96% |
| *mbc^c1^/TM3* | 1 | 5 | 5 | 100% |
| *mbc^c1^* | 5 | 25 | 22 | 88% |
| *loner^T1032^/TM3* | 1 | 5 | 5 | 100% |
| *loner^T1032^* | 5 | 20 | 20 | 100% |
| *kette^J4-48^/TM3* | 1 | 5 | 5 | 100% |
| *kette^J4-48^* | 5 | 25 | 23 | 92% |
| *blow^1^/Cyo* | 1 | 5 | 5 | 100% |
| *blow^1^* | 4 | 25 | 25 | 100% |
| *sltr^S1946^/Cyo* | 1 | 5 | 5 | 100% |
| *sltr^S1946^* | 5 | 25 | 25 | 100% |
| *WASp^3D3-035^/TM3* | 1 | 10 | 10 | 100% |
| *WASp^3D3-035^* | 3 | 25 | 25 | 100% |
| *sltr^S1946^; kette^J4-48^* | 5 | 20 | 17 | 85% |
| *PHplcγ (adhere myoblast)* | 5 | 20 | 20 | 100% |

[1] No actin focus is formed in *sns* mutants, therefore, the number of actin focus examined is 0. We checked 25 potential FC-FCM contact sites, and did not see Dia enrichment.
