# Supplementary material for: The Formin Diaphanous Regulates Myoblast Fusion through Actin Polymerization and Arp2/3 Regulation
Source: PLoS Genet. 2015 Aug 21;11(8):e1005381. doi: 10.1371/journal.pgen.1005381 (PMC4546610; doi:10.1371/journal.pgen.1005381)
Supplement: S2 Table — (DOCX) [file pgen.1005381.s002.docx]

| genotype | Muscle phenotype at stage 16 |
| --- | --- |
| *dia^5^/dia^5^* | Wild-type 57.7%, missing muscle 13.5%, detachment 21.2%, lack of fusion 3.8% muscle morphology 3.8% |
| *dia^2^/dia^2^* | Wild-type 0%, muscle defects including missing muscles, free myoblasts, detachment and muscle morphology changes (100%) |
| *otu-Gal4; nos-Gal4; nos-Gal4 x UAS-dia-RNAi->F1x Dmef2-Gal4* | No eggs |
| *mat15-Gal4; mat67-Gal4 x UAS-dia-RNAi->F1 x Dmef2-Gal4* | 100% missing one LT muscle (off target effect of mat-Gal4) |
| *UAS-dicer; Dmef2-Gal4 > UAS-dia-RNAi(Trip 33424)* | 100% wild-type |
| *UAS-dicer; Dmef2-Gal4 > UAS-dia-RNAi(Trip 28541)* | 100% wild-type embryonic muscle  100% flightless adult fly |

Supplemental Table 2: muscle phenotype in *dia* mutants
