# Supplementary material for: The Formin Diaphanous Regulates Myoblast Fusion through Actin Polymerization and Arp2/3 Regulation
Source: PLoS Genet. 2015 Aug 21;11(8):e1005381. doi: 10.1371/journal.pgen.1005381 (PMC4546610; doi:10.1371/journal.pgen.1005381)
Supplement: S3 Table — (DOCX) [file pgen.1005381.s003.docx]

Table S3: Actin::GFP recovery rate and percentage of recovery in control and constitutively active Diaphanous embryos

| genotype | Half time of FRAP | | | Percentage recovery | | | N |
| --- | --- | --- | --- | --- | --- | --- | --- |
|  | min | max | average | min | max | average |  |
| *twi-actin::GFP, DMef2-Gal4 > oregon R*  *(control)* | 24.5 | 76.2 | 53.3±17.7 | 67.1 | 97.2 | 78.6±10.5 | 8 |
| *twi-actin::GFP; DMef2-Gal4 > UAS-dia.CA* | 5.7 | 28.9 | 16.2±6.7 | 66.0 | 101.6 | 77.9±10.6 | 10 |
