# Supplementary figures and images for: The Formin Diaphanous Regulates Myoblast Fusion through Actin Polymerization and Arp2/3 Regulation
Source: PLoS Genet. 2015 Aug 21;11(8):e1005381. doi: 10.1371/journal.pgen.1005381 (PMC4546610; doi:10.1371/journal.pgen.1005381)

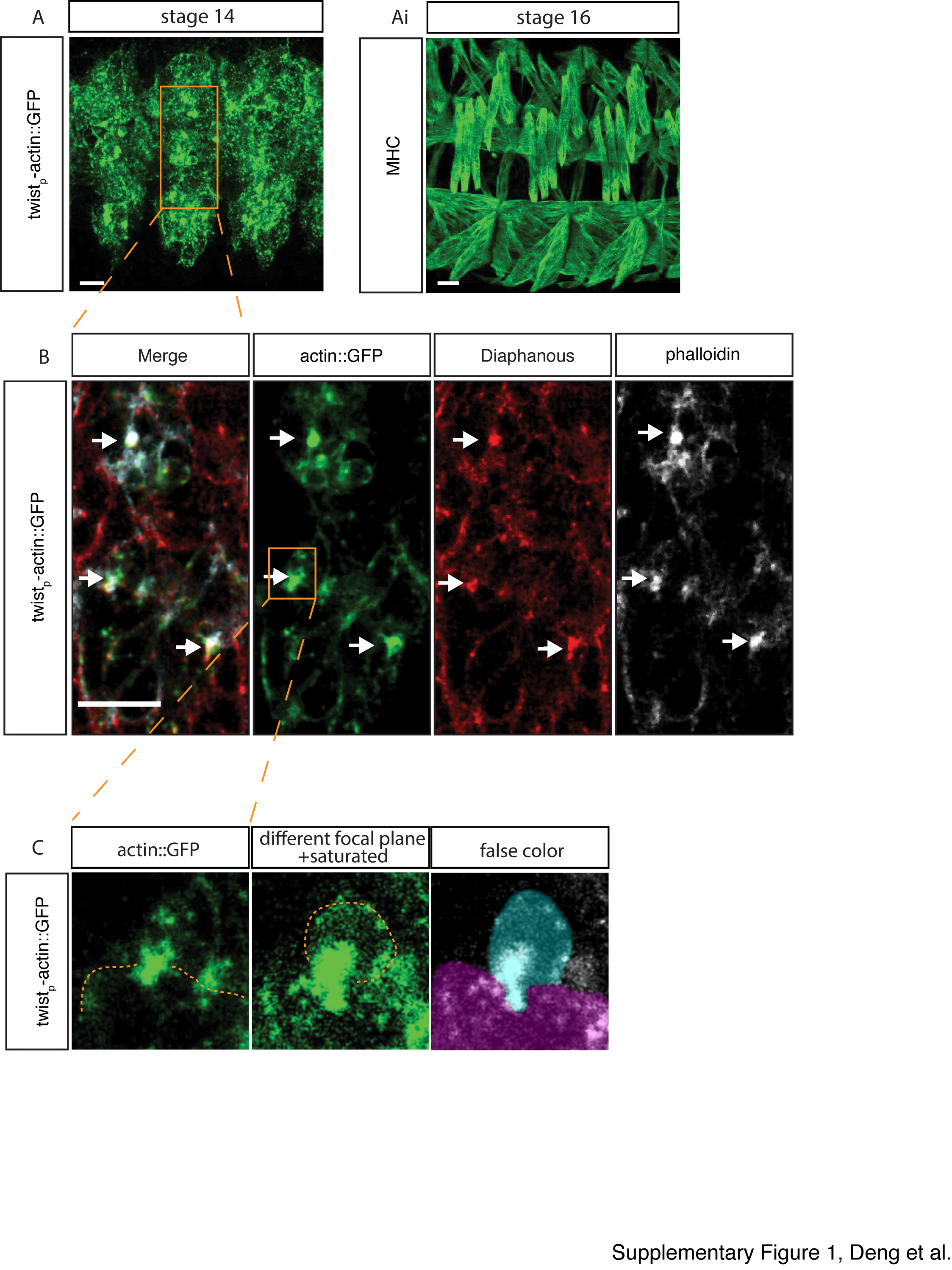

Supplement: S1 Fig — A. Projection of three hemisegments of a stage 14 twist-actin::GFP embryo stained for GFP which reveals the myoblasts in each hemisegments as fusion occurs. Ai. Three hemisegments of a stage 16 control embryo stained for MHC. This image reveals the muscle pattern that results from myoblast fusion. Scale bar: 10μM B. Higher magnification image showing single scan of the boxed area: one hemisegment from a stage 14 twist-actin::GFP embryo stained for F-actin (phalloidin) and antibodies against Dia and GFP. The F-actin focus at the fusion site was visualized both by GFP antibody (green; twist-actin::GFP) and phalloidin staining (white). Dia (red) is present in muscles and is enriched at fusion sites (arrows). Scale bar: 10μM. C. Cell outline determination used in all Figures: we determine the FCM and myotube cell outlines manually by adjusting brightness and changing focal planes. (TIF) [file pgen.1005381.s007.tif]

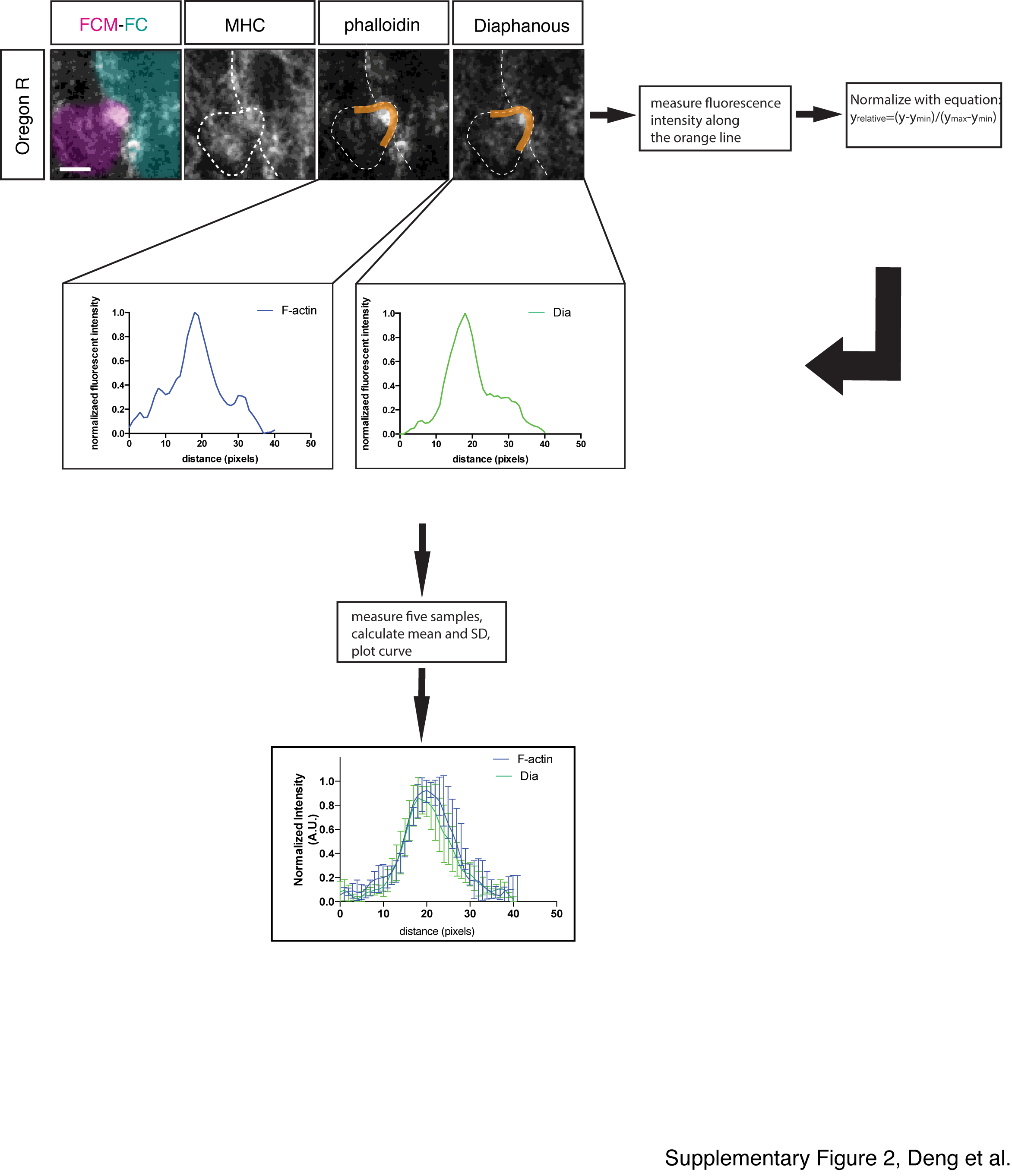

Supplement: S2 Fig — For all Figures except Fig 1: a line was drawn along cell cortex, with the center of the line localized at the actin focus or cell contact site. Fluorescent intensities were measured along the line and normalized using the equation shown in Figure. After the desired number of samples was measured, average relative intensities and standard deviations at each point were calculated and plotted. (TIF) [file pgen.1005381.s008.tif]

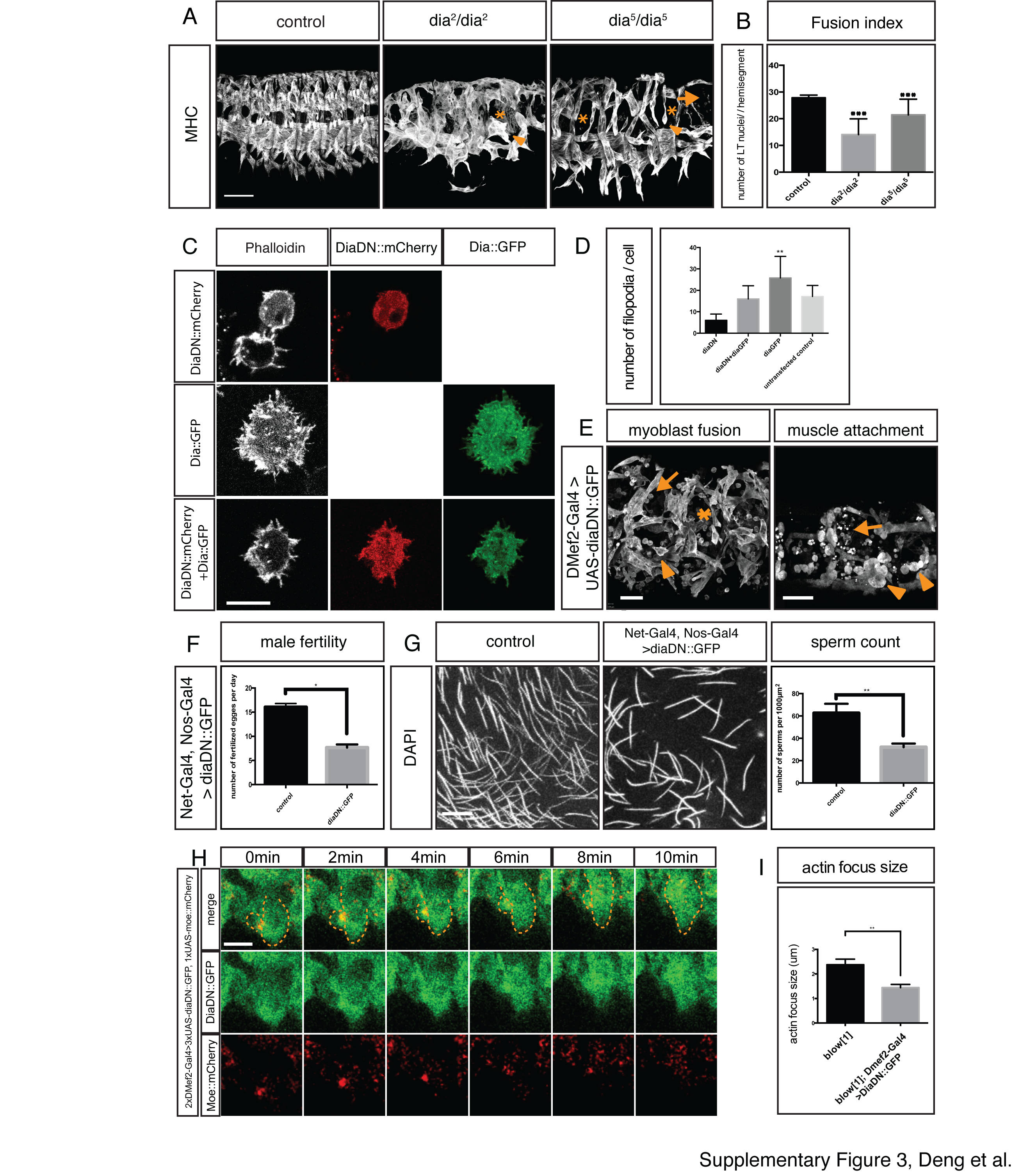

Supplement: S3 Fig — A. Stage 16 dia 2 and dia 5 homozygous embryos were stained with MHC to visualize muscle pattern. GFP antibody was used to identify balancer. dia 2 is an amorphic allele and dia 5 is a hypomorphic allele; both result in Dia loss of function. Homozygous dia mutants display a range of muscle defects, including muscle detachment (arrowhead), missing muscles (asterisk), free myoblasts (arrow), and muscle shape changes. Scale bar: 40μM. B. Fusion index of dia 2 and dia 5 homozygous mutants. apMe-NLS::dsRed was expressed in LT muscles to label nuclei, and fusion index was assessed in stage 17 embryos. Fusion was impaired in dia 2 and dia 5 homozygous mutant embryos (dia 2: 14.1±1.3, n = 21, dia 5: 21.5±1.3 n = 20 vs control: 27.8±0.3 n = 12, p<0.001). C. Expression of Dia::GFP rescued filopodia reduction by DiaDN. S2R+ cells that were co-transfected with Dia::GFP (green), and/or DiaDN::mCherry (red) (n = 7). Expression of DiaDN::mCherry results in a significant reduction of filopodia (phalloidin, grey in single channel). By contrast, expression of Dia::GFP resulted in increased cell spreading and increased numbers of filopodia (phalloidin, grey) compared to control. Expression of both constructs simultaneously results in more filopodia-like, protrusive structures relative to cells that are transfected with DiaDN::mCherry alone (n = 5). Scale bar: 10μM. D. Filopodia numbers were quantified in cells expressing DiaDN::mCherry, DiaDN::mCherry+Dia::GFP, Dia::GFP and mock treated control. E. Two copies of DMef2-Gal4 driving two copies of UAS-diaDN::GFP in myoblasts at 29°C. Antibodies to GFP were used to visualize the muscle cells. Embryonic muscle defects were found, including free myoblasts (arrow) and missing muscles (asterisk) at Stage 16 and severe muscle detachment (arrowhead) at Stage 17. Scale bar: 20μM and 40μM from left to right. F-G. In male flies which carry net-Gal4;nos-Gal4>UAS-diaDN::GFP, male fertility (F) and sperm number (G) were quantified and compared to con [file pgen.1005381.s009.tif]

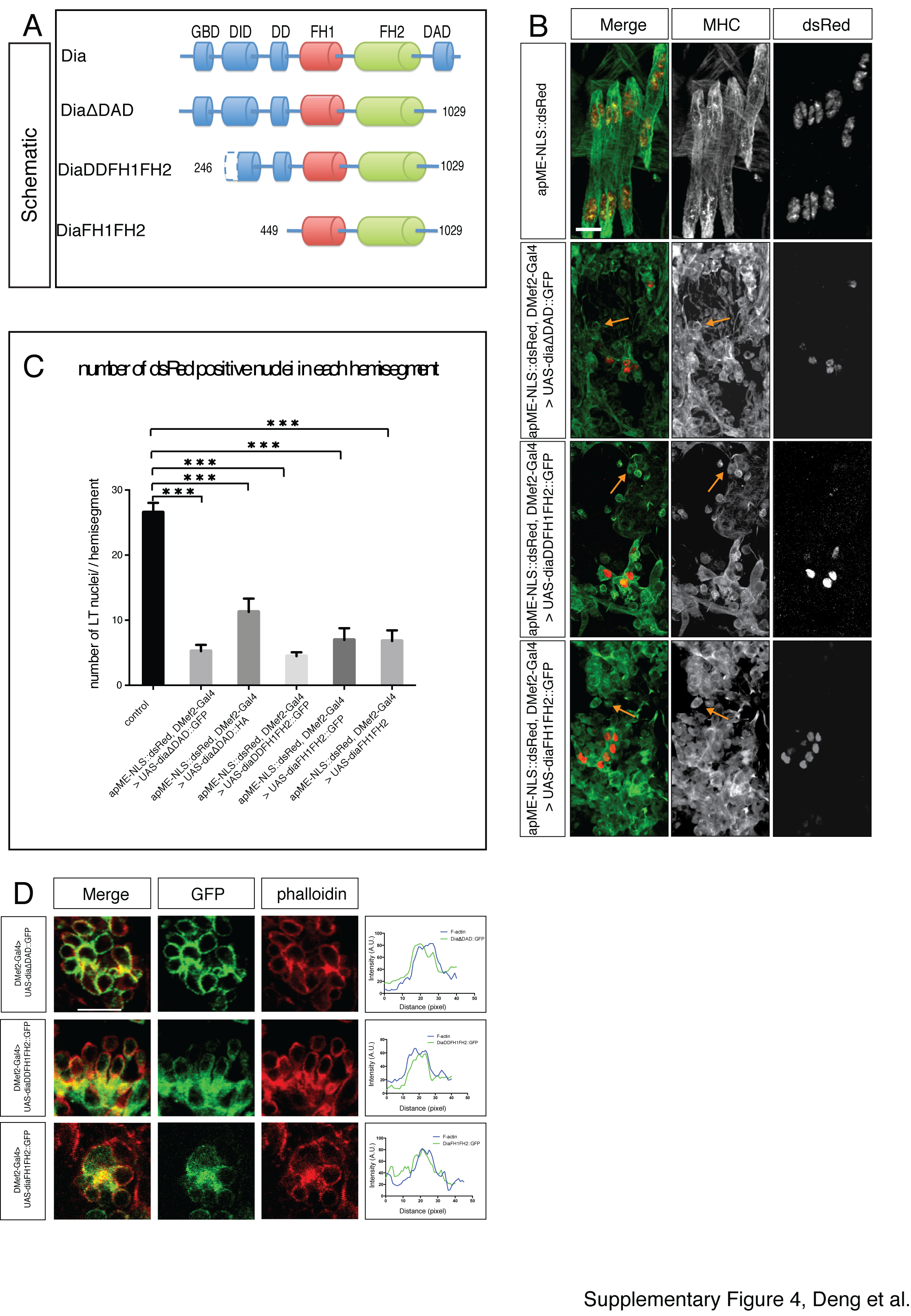

Supplement: S4 Fig — A. Schematic diagram of Dia domain structure and the different constitutively active Dia (DiaCA) deletion constructs (DiaΔDAD, diaDDFH1FH2, DiaFH1FH2) used in this study. B-C. Muscle pattern in a single hemisegment and Fusion index in embryos expressing the different DiaCA constructs. UAS-diaCA was expressed in muscles using the DMef2-Gal4 driver. MHC staining shows that myoblast fusion was blocked by DiaCA (high magnification of the LT Muscle area). To confirm this observation, apMe-NLS::dsRed was expressed in LT muscles to measure the fusion index. Compared with controls, all diaCA constructs significantly reduce myoblast fusion (graph in B. p<0.001). D. F-actin structure and DiaCA localization when expressing different diaCA constructs. F-actin was labeled with phalloidin. DiaCA localizations were visualized with GFP immunofluorescence staining. Instead of forming a defined actin focus at the fusion site, F-actin displayed a diffused localization in myoblasts expressing DiaCA. DiaDDFH1FH2 and DiaFH1FH2 both localize in the cytoplasm, while DiaΔDAD localizes primarily at the membrane. Intensity plot shows the colocalization of DiaCA (green) and actin structure (red) at the fusion site. Scale bars in B-D: 10μM. (TIF) [file pgen.1005381.s010.tif]

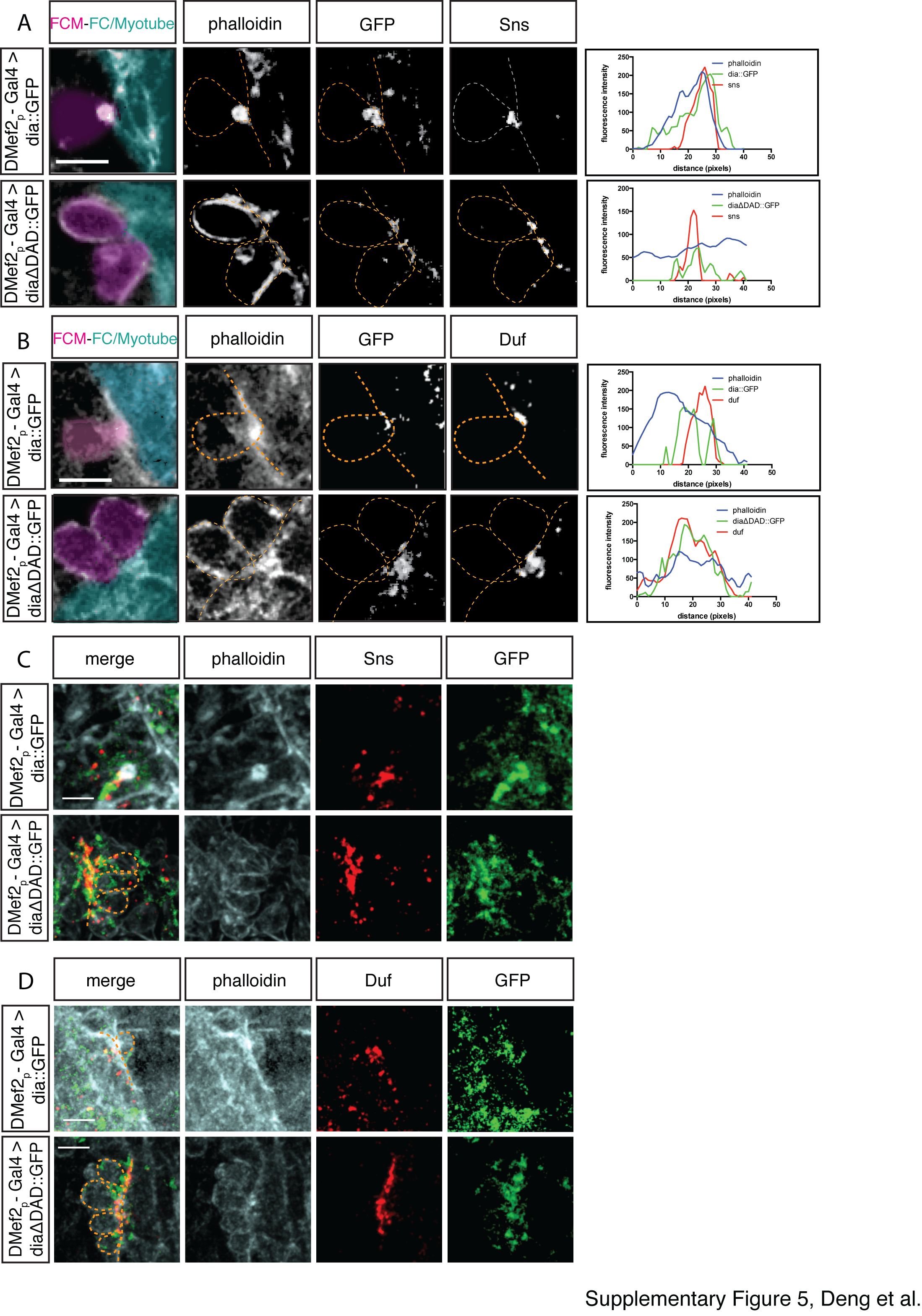

Supplement: S5 Fig — A. Sns localization in control (DMef2-Gal4>UAS-dia::GFP) and DMef2-Gal4>UAS-diaΔDAD::GFP embryos. Stage 14 embryos stained with phalloidin and antibodies against GFP and Sns. In control embryos, Sns is localized at the fusion site on the FCM side. Constitutively active Dia does not change Sns localization at the fusion site. Fluorescent intensity curves confirm that the Sns peak colocalizes with F-actin peak in both control and DMef2-Gal4>UAS-diaΔDAD::GFP embryos. B. Duf localization in control and DMef2-Gal4>UAS-diaΔDAD::GFP embryos. Stage 14 embryos stained with phalloidin and antibodies against GFP and Duf. In control embryos, Duf is localized at the fusion site on the FC/myotube side. Similar to Sns, Duf localization is not changed by DiaΔDAD::GFP. Fluorescent intensity curves confirm that the Duf peak colocalizes with the F-actin peak for both control and DMef2-Gal4/UAS-diaΔDAD::GFP embryos. The peak of Duf is broader and less defined in DMef2-Gal4>UAS-diaΔDAD::GFP embryos. C-D. Projection image of myoblasts expressing the constitutively active Dia construct DiaΔDAD. Actin (phalloidin, white); Dia (GFP antibody, green), Sns or Duf (antibody, Red). Dashed lines indicate FCMS adhered to the FC. Compared to the control embryo, the accumulation of Sns and Duf appears stronger due to an increased number of unfused and adhered myoblasts. Scale bar: 2.5μM. (TIF) [file pgen.1005381.s011.tif]
